# Laboulbeniales hyperparasites (Fungi, Ascomycota) of bat flies: Independent origins and host associations

**DOI:** 10.1002/ece3.4359

**Published:** 2018-07-24

**Authors:** Danny Haelewaters, Rachel A. Page, Donald H. Pfister

**Affiliations:** ^1^ Department of Organismic and Evolutionary Biology Farlow Reference Library and Herbarium of Cryptogramic Botany Harvard University Cambridge Massachusetts; ^2^ Smithsonian Tropical Research Institute Balboa Panama

**Keywords:** Ascomycota, ectoparasites, host specialization, phenotypic plasticity, ribosomal DNA, taxonomy

## Abstract

The aim of this study was to explore the diversity of ectoparasitic fungi (Ascomycota, Laboulbeniales) that use bat flies (Diptera, Hippoboscoidea) as hosts. Bat flies themselves live as ectoparasites on the fur and wing membranes of bats (Mammalia, Chiroptera); hence this is a tripartite parasite system. Here, we collected bats, bat flies, and Laboulbeniales, and conducted phylogenetic analyses of Laboulbeniales to contrast morphology with ribosomal sequence data. Parasitism of bat flies by Laboulbeniales arose at least three times independently, once in the Eastern Hemisphere (*Arthrorhynchus*) and twice in the Western Hemisphere (*Gloeandromyces*,* Nycteromyces*). We hypothesize that the genera *Arthrorhynchus* and *Nycteromyces* evolved independently from lineages of ectoparasites of true bugs (Hemiptera). We assessed phylogenetic diversity of the genus *Gloeandromyces* by considering the LSU rDNA region. Phenotypic plasticity and position‐induced morphological adaptations go hand in hand. Different morphotypes belong to the same phylogenetic species. Two species, *G. pageanus* and *G. streblae*, show divergence by host utilization. In our assessment of coevolution, we only observe congruence between the Old World clades of bat flies and Laboulbeniales. The other associations are the result of the roosting ecology of the bat hosts. This study has considerably increased our knowledge about bats and their associated ectoparasites and shown the necessity of including molecular data in Laboulbeniales taxonomy.

## 1 INTRODUCTION

Hyperparasitism, whereby parasites infect other parasites, is thought to be a common phenomenon in nature (Parratt & Laine, 2016). Few examples of obligate hyperparasites among fungi, however, have been well studied. Questions arise about what appears at first glance to be a risky lifestyle (Parratt, Barrès, Penczykowski, & Laine, 2017). How did such associations evolve? What population parameters are necessary to maintain these relationships? How strict are the species‐level relationships? The examples studied here involve bats, their blood‐sucking dipteran ectoparasites and the fungal ectoparasites of the blood‐sucking flies. An important question is whether this lifestyle could have arisen multiple times even though it seems tenuous. Another unexplored question is how diverse these fungal hyperparasites are, especially in the tropical regions (Arnold & Lutzoni, 2007).

Bats (Mammalia, Chiroptera) have received a great deal of attention due to their extraordinary morphological and ecological adaptations as well as their diversity in life history traits, qualities that make them ideal study organisms. Bats are parasitized by different groups of organisms, of which bat flies (Diptera, Hippoboscoidea, Nycteribiidae and Streblidae) are relatively well studied compared to other parasites. Published work has focused on host specificity, apparent male‐domination and population structure of bat flies (Dick & Patterson, 2007, 2008; Dittmar, Porter, Murray, & Whiting, 2006; Olival et al., 2013) and on associations between functional traits of bats and parasitism by bat flies (Patterson, Dick, & Dittmar, 2007). However, the addition of a second trophic level to the bat “microhabitat” is underexplored. Shockley and Murray (2006) reported two natural enemies of streblid bat flies (a hymenopteran parasitoid and a predaceous mirid bug). In addition, a handful of papers have discussed bacterial endosymbionts of bat flies in temperate and tropical regions (Duron et al., 2014; Hosokawa et al., 2012; Morse, Dick, Patterson, & Dittmar, 2012; Morse et al., 2013; Wilkinson et al., 2016).

In this study, we focus on the Laboulbeniales (Ascomycota, Laboulbeniomycetes), microscopic fungi that are obligate biotrophs on a wide range of arthropods, including bat flies. Prior to our current studies, the most recent papers dealing with Laboulbeniales on bat flies were published almost 40 years ago (Blackwell, 1980a, 1980b). Other papers on the same topic go back to the work of Harvard professor Roland Thaxter (1858–1932). Some of his publications presented species descriptions and new records for *Arthrorhynchus*, a genus apparently restricted to Old World bat flies (Thaxter, 1896, 1901, 1908, 1915, 1931), and two genera that thus far have only been reported on neotropical bat flies, *Gloeandromyces* and *Nycteromyces* (Thaxter, 1917, 1924, 1931). Until we initiated our studies on bat fly‐associated Laboulbeniales, five species were known from the type collections only (Haelewaters et al., 2017a, 2017b; Walker et al., 2018). This illustrates how underexplored these hyperparasites are. Windsor (1990, 1995) made the claim “Equal Rights for Parasites!” arguing that whereas parasites are generally either ignored or seen as a threat to conservation of endangered organisms, they should be recognized as a legitimate part of the earth's biodiversity. This applies as well to hyperparasites. All organisms are almost sure to acquire a parasite during their lifetime, even parasites themselves.

Laboulbeniales are one of three orders in the class Laboulbeniomycetes, the two others being Herpomycetales and Pyxidiophorales (Haelewaters et al., in review). All members of the class are obligately associated with arthropods for dispersal (Pyxidiophorales) or as biotrophs (Herpomycetales, Laboulbeniales). What sets the Laboulbeniales apart is its diversity, with 2,200 described species and many more awaiting discovery, and its wide variety of arthropod hosts. Representatives of three subphyla serve as hosts to Laboulbeniales: Chelicerata, with harvestmen (Opiliones) and mites (Acari); Myriapoda, with millipedes (Diplopoda); and Hexapoda, with cockroaches and termites (Blattodea), beetles (Coleoptera), earwigs (Dermaptera), flies (Diptera), true bugs (Hemiptera), ants (Hymenoptera, Formicidae), crickets and allies (Orthoptera), lice (Psocodea), and thrips (Thysanoptera). As ectoparasites, Laboulbeniales are attached to the exoskeleton of the host where they form multicellular units of determinate growth, or thalli. They are developmentally unique among the fungi that usually have mycelia of unlimited growth. Laboulbeniales thalli are the result of subsequent divisions of a single two‐celled ascospore. The ascospores are predominantly transmitted directly from infected to uninfected hosts (De Kesel, 1995).

Studying Laboulbeniales fungi has proven to be difficult for several reasons. The average size of Laboulbeniales thalli is around 200 μm, with extremes ranging from 35 μm (*Rickia depauperata* on mites of the genus *Celaenopsis*) to 4 mm (*Laboulbenia kunkelii* on *Mormolyce phyllodes* beetles). Because thalli are externally attached to a host, any study, morphological or molecular, requires micro‐manipulation with sterile techniques. Hosts may bear a large number of thalli, but often only few thalli are available for study. In some cases, thalli of a given species or morphotype may be restricted to a particular position on the host body (Goldmann & Weir, 2012; Goldmann, Weir, & Rossi, 2013). Unlike most fungi, Laboulbeniales have not been grown in culture to more than a few cells (never reaching maturity) (Whisler, 1968). The isolation of DNA has often been unsuccessful because of the often heavily pigmented cell walls (Weir & Blackwell, 2001b). This pigment, melanin, interferes during the PCR step by binding to the polymerase enzyme (Eckhart, Bach, Ban, & Tschachler, 2000). In addition, the cells are resilient in order to absorb impacts and friction on the host's integument. The combination of the melanized cell walls and resilient cells makes the thalli hard to break open.

Fungi of the order Laboulbeniales can display several types of specificity. Many species are host‐specific; they are associated with a single host species or species in the same genus. Based on experimental work, De Kesel (1996) showed that this specificity is driven by characteristics of the integument and living conditions of the arthropod host, but also by the habitat chosen by that host. For a number of species, such as *Euzodiomyces lathrobii*,* Hesperomyces virescens*,* Laboulbenia flagellata* and *Rhachomyces lasiophorus*, many host species are known, often in more than one host family (Santamaria, Balazuc, & Tavares, 1991). Our work with *H. virescens* has demonstrated that it is impossible to make accurate species‐level delimitations without molecular data (D. Haelewaters et al., unpublished data). It could be that more generalistic taxa are species complexes consisting of several species, whether cryptic or not, segregated by host. A different scenario is posed when hosts co‐occur in a single micro‐habitat. In this situation, opportunities exist for ascospores to be transmitted from a “typical” host to an “atypical” one. Such micro‐habitats might be ant nests (Pfliegler, Báthori, Haelewaters, & Tartally, 2016), subterranean caves (Reboleira, Fresneda, & Salgado, 2017), or seaweed and plant debris on beaches (De Kesel & Haelewaters, 2014). Another type of specificity is displayed when a given fungus shows “a remarkable tendency to grow on very restricted portions of the host integument” (Benjamin & Shanor, 1952). This phenomenon is referred to as position specificity. For example, 13 species of *Chitonomyces* can be observed on restricted positions of the aquatic diving beetle *Laccophilus maculosus*. Based on the combination of molecular and ecological data, Goldmann and Weir (2012) confirmed that sexual transmission is the mechanism behind the observed position specificity patterns, as suggested by Benjamin and Shanor (1952).

**Figure 1:**
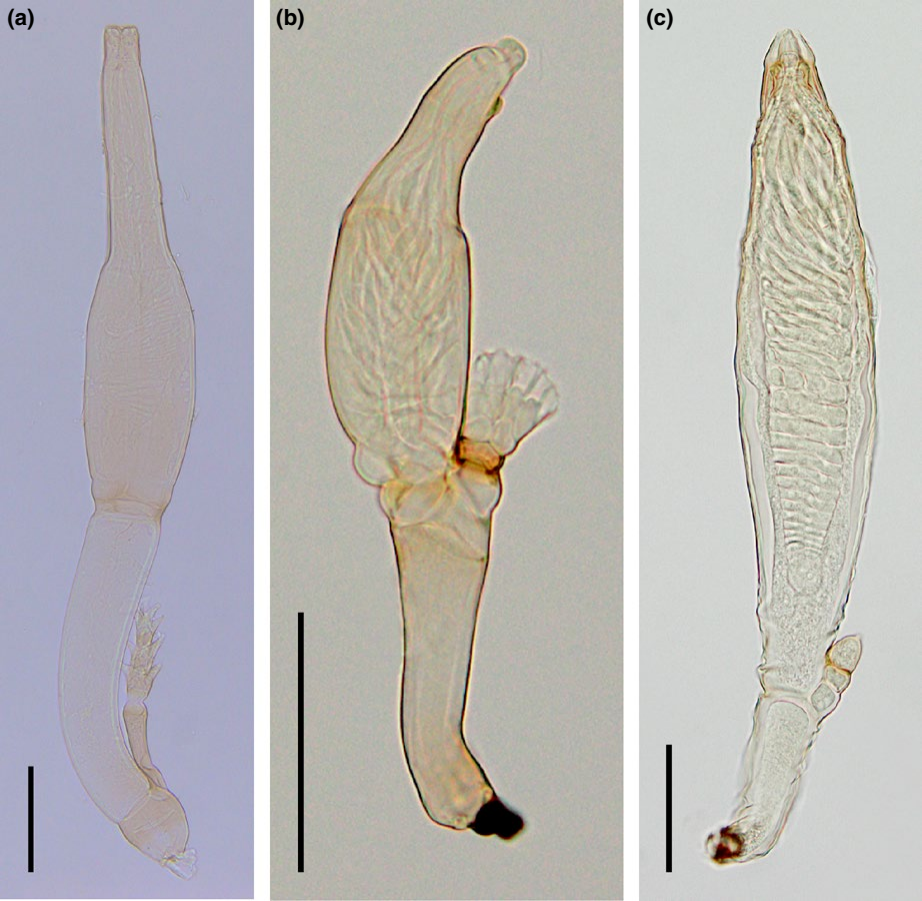
(a) *Arthrorhynchus nycteribiae*. (b) *Gloeandromyces streblae*. (c) *Nycteromyces streblidinus*, a female thallus

Around 10% of Laboulbeniales parasitize flies (Diptera). Species of Laboulbeniales on flies belong to eight genera: *Arthrorhynchus*,* Dimeromyces*,* Gloeandromyces*,* Ilytheomyces*,* Laboulbenia*,* Nycteromyces*,* Rhizomyces*, and *Stigmatomyces*. The genus *Laboulbenia* is by far the largest genus with over 800 species epithets listed in Index Fungorum (2018), but only 24 *Laboulbenia* species are known from flies (Rossi & Kirk‐Spriggs, 2011). *Stigmatomyces* is the second‐largest genus in the order, with 144 described species, all on flies (Rossi & Leonardi, 2013). The genera *Arthrorhynchus*,* Gloeandromyces* and *Nycteromyces* (Figure 1) are specific to bat flies, whereas none of the other genera have been recorded from bat flies.

*Arthrorhynchus* is restricted to Old World species of Nycteribiidae. Kolenati (1857) was the first to report Laboulbeniales from bat flies; he described two species, *Arthrorhynchus diesingii* from *Nycteribia vexata* [as *Acrocholidia montguei* (*vexata*)] and *A. westrumbii* from *Penicillidia conspicua* [as *Megistopoda westwoodii*]. Peyritsch (1871) described *Laboulbenia nycteribiae* and suggested that Kolenati's species were synonyms of his newly described taxon. He later erected a new genus to accommodate his species: *Helminthophana nycteribiae* (Peyritsch, 1873). Thaxter (1896) followed Peyritsch's opinion but later he (Thaxter, 1901) retained *Arthrorhynchus* and described two additional species, *A. cyclopodiae* and *A. eucampsipodae*. Another species, *A. acrandros*, was described by Merola (1952) from the bat fly *Phthiridium biarticulatum* [as *Nycteribia* (*Celepries*) *biarticulata*]. The taxonomic status of all these species is unclear, because no sequence data exist for any of them (except *A. nycteribiae*). *Arthrorhynchus nycteribiae* has been reported from several host genera: *Nycteribia*,* Penicillidia*,* Phthiridium* (Blackwell, 1980b). Consequently, this taxon could be a complex of different species, each specialized to a single bat fly host or several hosts in a single genus—as is the situation in *Hesperomyces virescens* (D. Haelewaters et al., unpublished data).

The genera *Gloeandromyces* and *Nycteromyces* have hitherto only been found on streblid bat flies in the Americas (Haelewaters et al., 2017b; Thaxter, 1917, 1931; Walker et al., 2018). The diversity of both genera is thus far limited, as is knowledge of their distribution and biology. After their original description (Thaxter, 1917), *G. nycteribiidarum*,* G. streblae* [both described as *Stigmatomyces*] and *Nycteromyces streblidinus* were not reported again until a century later by Haelewaters et al. (2017b). *Gloeandromyces nycteribiidarum* was described on *Megistopoda aranea* [as *Pterellipsis aranea*] from Grenada, and *G. streblae* on *Strebla wiedemanni* [as *S. vespertilionis*] from Venezuela. *Nycteromyces streblidinus* was described on the same individual of *S. wiedemanni* from which *G. streblae* had been described (Thaxter, 1917). Haelewaters et al. (2017b) described a third species of *Gloeandromyces*,* G. pageanus*, from *Trichobius dugesioides* bat flies collected in Gamboa, Panama.

Except for a few disparate records of bat fly‐associated Laboulbeniales, virtually nothing is known about this triparatite system. Bat flies are dependent on their bat hosts (Ramasindrazana, Goodman, Gomard, Dick, & Tortosa, 2017) and it has been shown that habitat disturbance affects parasitism of bats by bat flies (Pilosof, Dick, Korine, Patterson, & Krasnov, 2012). The direction of the correlation (positive or negative) was reliant on the bat host species. Similarly, life history traits of both bats and bat flies may affect the ecology of Laboulbeniales species. If bat flies are affected by habitat disturbance, then Laboulbeniales species could be affected as well. For example, elevated population densities of bat flies would potentially increase transmission success of ascospores if they co‐occur on the same bat hosts or in the same roosts. However, for these sorts of data, hundreds or even thousands of bat flies need to be collected and screened for parasitic fungi. How life history traits and environmental factors such as habitat modification can shape species responses remains poorly understood and requires a large, non‐biased dataset. Toward this end, our main intentions were to collect and screen large numbers of bat flies, both through our own field collections and by expanding our network of collaborators who could provide us with bat flies.

## 2 MATERIALS AND METHODS

### 2.1 Capture of bats and collection of bat flies

**Figure 2:**
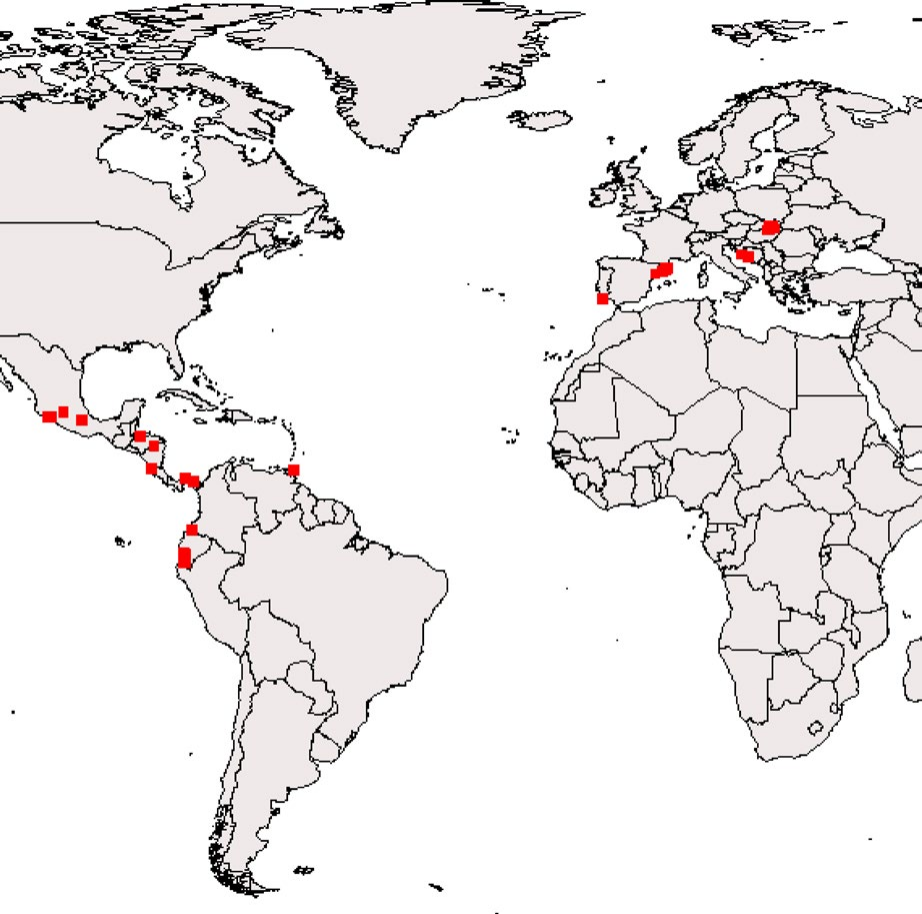
Field sites where bat flies for this project have been collected. Field sites are located in North and Central America (Costa Rica, Honduras, Mexico, Nicaragua, Panama), South America (Ecuador, Trinidad), and Europe (Croatia, Hungary, Slovakia, Portugal, Spain)

Bats were captured and screened for ectoparasites by D.H. with the help of collaborators and field assistants during several field trips to Panama between 2015 and 2017. Field sites were located at Isla Barro Colorado (Panamá Oeste Province); in Gamboa and Parque Nacional Soberanía in the Canal Zone (Colón Province); Chilibre (Panamá Province); and Reserva Natural Chucantí (Darién Province) (Figure 2). Bats were captured using three to four 6 m‐wide 36 mm mesh ground‐level mistnets with four shelves (Avinet, Portland, Maine, USA). Mistnets were set over trails that were presumably used by bats as flight pathways (Palmeirim & Etherdige, 1985). Nets were usually examined every 10–20 min between sunset and ~11 p.m. Bats were disentangled and processed immediately or kept in clean cotton bags until processing. Bats were released at the capture site immediately after processing. Bats were identified on site using dichotomous keys (Handley, 1981; Timm & LaVal, 1998). Bat taxonomy follows Simmons (2005). In this study, *Artibeus intermedius* was considered a junior synonym of *A. lituratus* (Barquez, Perez, Miller, & Diaz, 2015; Guerrero et al., 2008).

To remove bat flies from their bat hosts, 99% ethanol was applied using a paintbrush to reduce their activity. Subsequently, the bat flies were carefully removed using a rigid Swiss Style Forceps #5 with superfine tip (BioQuip #4535, Rancho Dominguez, California) or a Featherweight Forceps with narrow tip (BioQuip #4748). Some bat flies were collected using forceps alone or simply by hand. Preservation and long‐term storage of bat flies was in 99% ethanol in separate vials (one vial per bat host). Identification of bat flies to species level was based on published keys (Guerrero, 1993, 1994a, 1994b, 1995a, 1995b, 1996, 1997, 1998a, 1998b; Wenzel, 1976; Wenzel & Tipton, 1966) and complementary publications (Dick, 2013; Miller & Tschapka, 2001). Voucher specimens will be deposited at the following locations: Museo de Peces de Agua e Invertebrados, David, Panamá (MUPADI) and Naturalis Biodiversity Center, Leiden, Netherlands (RMNH). Labels for infected bat flies can be found in the Supporting Information.

Additional bat fly specimens preserved in 70%–99% ethanol were available from fieldwork by collaborators. Included in this study were bat flies from Latin America [Costa Rica (T. Hiller, unpublished data), Ecuador, Mexico, Nicaragua (C.W. Dick, unpublished data), Honduras (Dick, 2013), Panama (Walker et al., 2018) and Trinidad (J.J. Camacho, unpublished data)] and Europe [Croatia, Hungary, Portugal, Slovakia, and Spain in Europe (Haelewaters et al., 2017a; Szentiványi et al., 2018)].

### 2.2 Collection and identification of Laboulbeniales

Bat flies were screened for the presence of Laboulbeniales thalli under a Zeiss Stemi 508 stereomicroscope (Thornwood, New York). Thalli were removed from the host at the point of attachment (foot or haustorium) using Minuten Pins (BioQuip #1208SA, Rancho Dominguez, California) inserted onto wooden rods. Following Benjamin's (1971) procedure, thalli or groups of thalli were removed and mounted in Amann's medium, a liquid solution. Before applying Amann's medium and to facilitate microscopic observations, thalli first had to be arranged and fixed onto the microscope slide. To make thalli a bit sticky, they were first placed in a droplet of Hoyer's medium (30 g arabic gum, 200 g chloral hydrate, 16 ml glycerol, 50 ml ddH_2_O). Next, thalli were individually picked up and arranged in one or two rows. After a brief period of drying, the slide was closed using a cover slip with a drop of Amann's medium (drop facing downward) and subsequently sealed with nail polish or B‐72 in acetone (Gaylord #AB72, Syracuse, New York). Mounted specimens were viewed at 400× to 1,000× magnification under an Olympus BX53 compound microscope equipped with an Olympus DP73 digital camera (Waltham, Massachusetts). For detailed morphological study and descriptions at the Farlow Herbarium an Olympus BX40 microscope with XC50 camera was used. Fungal specimens were identified using Thaxter (1917, 1924, 1931) and Haelewaters et al. (2017b). Voucher slides are deposited at Farlow Herbarium (FH; Harvard University, Cambridge, Massachusetts) and Herbario de la Universidad Autónoma de Chiriquí (UCH; David, Panamá).

### 2.3 DNA extraction, PCR amplification, sequencing

DNA was extracted from 1–14 Laboulbeniales thalli using the Extract‐N‐Amp Plant PCR Kit (Sigma‐Aldrich, St. Louis, Missouri) (Haelewaters et al., 2015) or the REPLI‐g Single Cell Kit (Qiagen, Valencia, California) (Haelewaters et al., in review). Pretreatments employed with the Extract‐N‐Amp method included a prolonged incubation period at 56°C in 20 μl Extraction Solution up to 24‐hr in a Shake ‘N Bake Hybridization Oven (Boekel Scientific model #136400‐2, Feasterville, Pennsylvania) and mechanically crushing fungal material in a FastPrep FP120 Cell Disrupter (Thermo Fisher Scientific, Waltham, Massachusetts) at 5.5 m/s for 20 s. For about two thirds of our extractions, and as a rule for later extractions, thalli were manually cut in 2 or 3 parts (usually through the perithecium) using a #10 surgical blade on disposable Bard‐Parker handle (Aspen Surgical, Caledonia, Michigan) to ensure successful lysis.

The nuclear small and large ribosomal subunits of the ribosomal DNA (SSU and LSU rDNA) were amplified. Primer pairs for SSU were NSL1 (5′‐GTAGTGTCCTCrCATGCTTTTGAC‐3′) and NSL2 (5′‐AATCyAAGAATTTCACCTCTGAC‐3′) or NSL1 and R (5′‐TGATCCTTCTGCAGGTTCACCTACG‐3′) (Haelewaters et al., 2015; Wrzosek, 2000). Primer pairs for LSU were LR0R (5′‐ACCCGCTGAACTTAAGC‐3′) and LR5 (5′‐ATCCTGAGGGAAACTTC‐3′) or LIC24R (5′‐GAAACCAACAGGGATTG‐3′) and LR3 (5′‐GGTCCGTGTTTCAAGAC‐3′) (Miadlikowska & Lutzoni, 2000, Vilgalys & Hester, 1990; R. Vilgalys, unpublished data). PCR reactions consisted of 13.3 μl of RedExtract Taq polymerase (Sigma‐Aldrich), 2.5 μl of each 10 μM primer, 5.7 μl of H_2_O and 1.0 μl of template DNA. All amplifications were done in a 2720 Thermal Cycler (Applied Biosystems, Foster City, California) with initial denaturation at 94°C for 3:00 min; followed by 35 cycles of denaturation at 94°C for 1:00 min, annealing at 50°C for 0:45 min and extension at 72°C for 1:30 min; and final extension at 72°C for 10:00 min.

Unsuccessful PCR reactions were re‐run using the Q5 Host Start High‐Fidelity DNA Polymerase (New England BioLabs, Ipswich, Massachusetts). PCR was done in 25 μl consisting of 5.0 μl of 5× Q5 Reaction Buffer, 0.5 μl of 10 mM dNTP Mix (Quantabio, Beverly, Massachusetts), 1.25 μl of each 10 μM primer, 0.25 μl of Q5 High‐Fidelity DNA Polymerase, 12.75 μl of H_2_O and 4.0 μl of template DNA. Thermal cycling conditions were as follows: initial denaturation at 98°C for 30 s; 35 cycles of denaturation at 98°C for 10 s, annealing at 58–61.5°C for 30 s and extension at 72°C for 30 + 5/cycle s; followed by final extension at 72°C for 2 min. The optimal annealing temperature (Ta) was calculated for every primer combination using the New England BioLabs online Tm Calculator tool (tmcalculator.neb.com/) selecting “Q5” as product group and “Q5 Hot Start High‐Fidelity DNA Polymerase” as polymerase/kit, and with 500 mM for primer concentration. When smears or weak bands were observed on gel, conditions were optimized to include multiple annealing temperatures: 98°C for 3 min; 30 cycles at 98°C for 10 s, 65–68.5°C for 30 s (decreasing 1°C every three cycles) and 72°C for 1:30 min; then 30 cycles at 98°C for 10 s, 58–61.5°C for 30 s and 72°C for 1:30 min; and a final extension step of 72°C for 2 min.

Molecular work was done both at the Molecular Multi‐User's Lab at the Naos Marine Laboratories (Smithsonian Tropical Research Institute, Panama) and at the Harvard University Herbaria (Cambridge, Massachusetts). The protocol was identical except for purification and sequencing. In Panama, PCR products were purified using the QIAquick PCR Purification Kit (Qiagen). Subsequently, 10 μl reactions were prepared with the same primers and 3.0 μl of purified PCR product. Sequencing reactions were performed using the Big Dye^®^ Terminator v3.1 Cycle Sequencing Kit (Life Technologies, Carlsbad, California). In Cambridge, purification and sequencing steps were outsourced to Genewiz (South Plainfield, New Jersey). Generated sequences were assembled and edited in Sequencher 4.10.1 (Gene Codes Corporation, Ann Arbor, Michigan). All sequences have been deposited in GenBank (accession numbers in Table 1).

**Table 1:** Overview of Laboulbeniomycetes sequences used in this study. Species names are listed for all isolates, with their hosts and country

Genus	Species	Host	Country	Isolate	Extraction protocol	# thalli used	SSU	LSU
*Arthrorhynchus*	*nycteribiae*	*Penicillidia conspicua*	Hungary	Edeleny_ 13.xi.2014	Heat extraction	4–5	KY094496	KY094497
*Arthrorhynchus*	*nycteribiae*	*Penicillidia conspicua*	Hungary	D. Haelew. 1015d	ExNA	7	MG438336	MG438363
*Camptomyces*	sp. nov.	*Astenus* sp.	Tanzania	D. Haelew. 1222d	REPLI‐g	1	MF314140	MF314141
*Gloeandromyces*	sp. nov. 3	*Trichobius joblingi*	Panama	D. Haelew. 1312b	REPLI‐g, crushed	2	**MH040546**	**MH040580**
*Gloeandromyces*	sp. nov. 3	*Trichobius joblingi*	Panama	D. Haelew. 1312c	REPLI‐g	2	**MH040547**	**MH040581**
*Gloeandromyces*	sp. nov. 3	*Trichobius joblingi*	Panama	D. Haelew. 1323b	REPLI‐g, crushed	4	MG958011	**MH040582**
*Gloeandromyces*	sp. nov. 3	*Trichobius joblingi*	Panama	D. Haelew. 1323c	REPLI‐g, crushed	4	**MH040548**	**MH040583**
*Gloeandromyces*	*nycteribiidarum*	*Megistopoda aranea*	Panama	D. Haelew. 1319b	REPLI‐g	2	**MH040533**	**MH040566**
*Gloeandromyces*	*nycteribiidarum*	*Megistopoda aranea*	Panama	D. Haelew. 1334c	REPLI‐g, crushed	3	**MH040534**	**MH040567**
*Gloeandromyces*	sp. nov. 1	*Trichobius joblingi*	Panama	D. Haelew. 1306b	REPLI‐g	2	**MH040541**	**MH040574**
*Gloeandromyces*	sp. nov. 1	*Trichobius joblingi*	Panama	D. Haelew. 1322a	REPLI‐g, crushed	1	**MH040543**	**MH040577**
*Gloeandromyces*	sp. nov. 1	*Trichobius joblingi*	Panama	D. Haelew. 1327a	REPLI‐g, crushed	1	**MH040544**	**MH040578**
*Gloeandromyces*	sp. nov. 4	*Trichobius joblingi*	Trinidad	D. Haelew. 619a	ExNA	12	**MH040537**	KT800008
*Gloeandromyces*	sp. nov. 4	*Trichobius joblingi*	Panama	D. Haelew. 1073b	ExNA, prolonged, crushed	3	**MH040538**	**MH040570**
*Gloeandromyces*	sp. nov. 4	*Trichobius dugesioides*	Panama	D. Haelew. 1089a	ExNA, prolonged, crushed	4	**MH040539**	**MH040571**
*Gloeandromyces*	sp. nov. 4	*Trichobius joblingi*	Panama	D. Haelew. 1100b	ExNA, prolonged, crushed	7	**MH040307**	**MH040572**
*Gloeandromyces*	sp. nov. 3	*Trichobius dugesioides*	Panama	D. Haelew. 1272a	REPLI‐g, crushed	2	**MH040540**	**MH040573**
*Gloeandromyces*	sp. nov. 4	*Trichobius joblingi*	Panama	D. Haelew. 1315a	REPLI‐g, crushed	1	—	**MH040575**
*Gloeandromyces*	sp. nov. 4	*Trichobius joblingi*	Panama	D. Haelew. 1315b	REPLI‐g	2	**MH040542**	**MH040576**
*Gloeandromyces*	*pageanus*	*Trichobius dugesioides*	Panama	D. Haelew. 1091b	ExNA, prolonged, crushed	6	**MH040535**	MG906798
*Gloeandromyces*	*pageanus*	*Trichobius dugesioides*	Panama	D. Haelew. 1367b	EXNA, crushed, FastPrep	6	—	**MH040568**
*Gloeandromyces*	*pageanus*	*Trichobius dugesioides*	Panama	D. Haelew. 1425a	REPLI‐g, crushed	4	**MH040536**	**MH040569**
*Gloeandromyces*	*streblae*	*Trichobius dugesioides*	Panama	D. Haelew. 1090a	ExNA, prolonged, crushed	7	—	**MH040584**
*Gloeandromyces*	*streblae*	*Trichobius joblingi*	Panama	D. Haelew. 1306c	REPLI‐g	4	MG958012	**MH040585**
*Gloeandromyces*	*streblae*	*Trichobius dugesioides*	Panama	D. Haelew. 1308b	REPLI‐g	2	**MH040549**	**MH040586**
*Gloeandromyces*	*streblae*	*Trichobius dugesioides*	Panama	D. Haelew. 1309a	REPLI‐g	1	**MH040550**	**MH040587**
*Gloeandromyces*	*streblae*	*Trichobius joblingi*	Panama	D. Haelew. 1317a	REPLI‐g	1	**MH040551**	**MH040588**
*Gloeandromyces*	*streblae*	*Trichobius joblingi*	Panama	D. Haelew. 1335c	REPLI‐g, crushed	2	**MH040552**	**MH040589**
*Gloeandromyces*	sp. nov. 2	*Trichobius joblingi*	Panama	D. Haelew. 1320b	REPLI‐g, crushed	1	**MH040545**	**MH040579**
*Herpomyces*	*chaetophilus*	*Periplaneta americana*	USA	D. Haelew. 483b	ExNA	11 fem	MG438319	MG438350
*Herpomyces*	*chaetophilus*	*Periplaneta americana*	USA	D. Haelew. 602b	ExNA	10 fem	KT800023	KT800009
*Herpomyces*	*periplanetae*	*Periplaneta americana*	USA	D. Haelew. 602d	ExNA	8 fem	MG438327	MG438357
*Herpomyces*	*periplanetae*	*Periplaneta americana*	USA	D. Haelew. 1187d	REPLI‐g	1 fem	MG438331	MG438359
*Herpomyces*	*shelfordellae*	*Shelfordella lateralis*	Hungary	DE_HerpBL1	Heat extraction	±30	KT800026	KT800011
*Herpomyces*	*shelfordellae*	*Shelfordella lateralis*	Hungary	Bud_Slat	Heat extraction	10–20	MG438333	MG438361
*Herpomyces*	*stylopygae*	*Blatta orientalis*	Hungary	Bud_Bori	Heat extraction	10–20	MG438332	MG438360
*Hesperomyces*	*coleomegillae*	*Coleomegilla maculata*	Ecuador	631C	0.1 × TE buffer + dry ice	3–15	KF266882	—
*Hesperomyces*	*coleomegillae*	*Coleomegilla maculata*	Ecuador	632A	0.1 × TE buffer + dry ice	3–15	KF266880	—
*Hesperomyces*	*palustris*	*Coleomegilla maculata*	Ecuador	631K	0.1 × TE buffer + dry ice	3–15	KF266902	—
*Hesperomyces*	*palustris*	*Coleomegilla maculata*	Ecuador	632B	0.1 × TE buffer + dry ice	3–15	KF266891	—
*Hesperomyces*	*virescens*	*Harmonia axyridis*	USA	D. Haelew. 316a	ExNA	10–12	MG438339	KJ842339
*Hesperomyces*	*virescens*	*Harmonia axyridis*	Netherlands	D. Haelew. 334b	ExNA	10	MG438340	MG438364
*Hesperomyces*	*virescens*	*Olla v‐nigrum*	USA	JP352b	ExNA	11	MG760581	MG745337
*Hesperomyces*	*virescens*	*Olla v‐nigrum*	USA	JP353a	QIAamp Micro	10	KT800028	KT800013
*Hesperomyces*	*virescens*	*Olla v‐nigrum*	USA	JP354b	ExNA	10	MG760583	MG745339
*Hesperomyces*	*virescens*	*Harmonia axyridis*	South Africa	D. Haelew. 648c	ExNA	8–10	KU574863	KU574865
*Hesperomyces*	*virescens*	*Cheilomenes propinqua*	South Africa	D. Haelew. 655c	ExNA	11	KU574866	KU574867
*Hesperomyces*	*virescens*	*Cheilomenes propinqua*	South Africa	D. Haelew. 659a/b	ExNA	20	MG760590	MG745342
*Hesperomyces*	*virescens*	*Harmonia axyridis*	Netherlands	D. Haelew. 1174a	ExNA, crushed, prolonged	12	MG760598	MG745345
*Hesperomyces*	*virescens*	*Adalia bipunctata*	Denmark	D. Haelew. 1193g	REPLI‐g, crushed	1	MG760599	MG745346
*Hesperomyces*	*virescens*	*Adalia bipunctata*	Sweden	D. Haelew. 1199h	REPLI‐g, crushed	1	MG760600	MG745347
*Hesperomyces*	*virescens*	*Olla v‐nigrum*	USA	D. Haelew. 1200i	REPLI‐g, crushed	4	MG760602	MG745349
*Hesperomyces*	*virescens*	*Adalia bipunctata*	Italy	D. Haelew. 1231a	REPLI‐g	2	MG760603	MG745350
*Hesperomyces*	*virescens*	*Psyllobora vigintimaculata*	USA	D. Haelew. 1250b	REPLI‐g	5	MG760607	MG745354
*Hesperomyces*	*virescens*	*Psyllobora vigintimaculata*	USA	D. Haelew. 1250c	REPLI‐g, crushed	2	MG760608	MG745355
*Hesperomyces*	*virescens*	*Psyllobora vigintimaculata*	USA	D. Haelew. 1251b	REPLI‐g, crushed	1	MG760609	MG745356
*Hesperomyces*	*virescens*	*Harmonia axyridis*	Japan	D. Haelew. 1268b	REPLI‐g, crushed	3	MG760610	MG745357
*Nycteromyces*	*streblidinus*	*Trichobius parasiticus*	Honduras	D. Haelew. 956a	ExNA	8 fem	**MH040553**	—
*Nycteromyces*	*streblidinus*	*Trichobius joblingi*	Panama	D. Haelew. 1324b	REPLI‐g, crushed	4 m	**MH040554**	**MH040590**
*Nycteromyces*	*streblidinus*	*Trichobius joblingi*	Panama	D. Haelew. 1324c	REPLI‐g, crushed	1 fem	**MH040555**	—
*Nycteromyces*	*streblidinus*	*Trichobius joblingi*	Panama	D. Haelew. 1324d	REPLI‐g	1 fem	**MH040556**	**MH040591**
*Nycteromyces*	*streblidinus*	*Trichobius joblingi*	Panama	D. Haelew. 1324e	REPLI‐g, crushed	1 m	**MH040557**	**MH040592**
*Polyandromyces*	*coptosomalis*	*Phoeacia* sp. nov.	Ecuador	D. Haelew. 313f	ExNA	7 fem, 2 m	KT800035	KT800020
*Polyandromyces*	*coptosomalis*	*Acrosternum* sp.	Canary Islands	HM499a	ExNA	15 fem, 3 m	MG438347	—
*Prolixandromyces*	*triandrus*	*Velia* (*Plesiovelia*) *saulii*	Hungary	Nagyvisnyo1	Heat extraction	5	LT158294	LT158295
*Rickia*	*laboulbenioides*	*Cylindroiulus punctatus*	Denmark	SR4s	ExNA, crushed	5	**MH040558**	**MH040593**
*Rickia*	*pachyiuli*	*Pachyiulus hungaricus*	Serbia	SR1s	ExNA, crushed	10–12	**MH040559**	**MH040594**
*Rickia*	*wasmannii*	*Myrmica scabrinodis*	Hungary	DE_Rak4	Heat extraction	30	KT800037	KT800021
*Rickia*	*wasmannii*	*Myrmica sabuleti*	Netherlands	D. Haelew. 1234a	REPLI‐g	3	**MH040560**	**MH040595**
*Stigmatomyces*	*borealis*	*Parydra breviceps*	USA	AW‐797			JN835186	—
*Stigmatomyces*	*ceratophorus*	*Fannia canicularis*	USA	D. Haelew. 1136h	REPLI‐g, crushed	8	MG958013	**MH145384**
*Stigmatomyces*	*entomophilus*	*Drosophila funebris*	Netherlands	D. Haelew. 1062c	ExNA, prolonged, crushed	6	MG958014	—
*Stigmatomyces*	*entomophilus*	*Drosophila funebris*	Netherlands	D. Haelew. 1063a	ExNA, prolonged, crushed	14	**MH040561**	—
*Stigmatomyces*	*gregarius*	Diopsidae sp.	Sierra Leone	D. Haelew. 1008a	ExNA	5	MG438348	—
*Stigmatomyces*	*gregarius*	Diopsidae sp.	Sierra Leone	D. Haelew. 1008b	ExNA	±10	**MH040562**	—
*Stigmatomyces*	*hydrelliae*	*Hydrellia* sp.			0.1 × TE buffer + dry ice	4–10	AF431757	—
*Stigmatomyces*	*limnophorae*	Muscidae sp.	USA	AW‐785	1% Triton 100	4–10	AF407576	—
*Stigmatomyces*	*protrudens*	Ephydridae sp.	USA	AW‐793	0.1 × TE buffer + dry ice	4–10	AF298232	AF298234
*Stigmatomyces*	*rugosus*	*Psilopa* sp.			0.1 × TE buffer + dry ice	4–10	AF431759	—
*Stigmatomyces*	*rugosus*	*Psilopa* sp.	Portugal	D. Haelew. 1138a	ExNA, prolonged, crushed	6	**MH040563**	—
*Stigmatomyces*	*scaptomyzae*	*Scaptomyza* sp.			0.1 × TE buffer + dry ice	4–10	AF431758	—
*Stigmatomyces*	sp. nov.	cf. *Chamaemyia*	Portugal	D. Haelew. 1137a	ExNA, prolonged, crushed	8	**MH040564**	—
*Stigmatomyces*	sp. nov.	cf. *Chamaemyia*	Portugal	D. Haelew. 1137c	ExNA, prolonged, crushed	1	**MH040565**	—

*Notes*. Also included are extraction protocols and numbers of thalli used per extraction for all isolates: 1% Triton 100‐based protocol from Weir and Blackwell (2001a); 0.1 × TE buffer + dry ice protocol from Weir and Blackwell (2001b); heat extraction protocol, Extract‐N‐Amp Plant PCR Kit (ExNA) and QIAamp DNA Micro Kit (QIAamp Micro) from Haelewaters et al. (2015); REPLI‐g Single Cell Kit (REPLI‐g) from Haelewaters et al. (in review). GenBank accession numbers are provided (newly generated sequences in bold).

### 2.4 Sequence alignment and phylogenetic analyses

SSU and LSU rDNA datasets were constructed of newly generated sequences and sequences downloaded from GenBank, in order to assess (a) the position of bat fly‐associated genera among Laboulbeniales from other hosts and (b) phylogenetic diversity in the genus *Gloeandromyces*. Alignments were done using Muscle v3.7 (Edgar, 2004) on the Cipres Science Gateway version 3.3 (Miller, Pfeiffer, & Schwartz, 2010) and manually edited in BioEdit v7.2.6 (Hall, 1999). The SSU and LSU aligned data matrices were concatenated in MEGA v7.0.21 (Kumar, Stecher, & Tamura, 2016). Maximum likelihood (ML) analysis of the SSU + LSU dataset was run using PAUP on XSEDE 4.0b (Swofford, 1991), which is available on Cipres. The appropriate nucleotide substitution model was selected by considering the Akaike Information Criterion (AIC) in jModelTest 2.1 (Darriba, Taboada, Doallo, & Posada, 2012). The general time reversible model (GTR) with the assumption of a gamma distribution (+G) gave the best scoring tree (−lnL = 15262.1769). ML was inferred under this model and bootstrap (BS) values were calculated with 200 replicates.

Bayesian analyses were run using the BEAST on XSEDE tool in Cipres with a Markov chain Monte Carlo (MCMC) coalescent approach, under an uncorrelated lognormal relaxed molecular clock model allowing rates of evolution to vary across the tree. The Birth‐Death Incomplete Sampling speciation model (Stadler, 2009) was selected as tree prior with the GTR+G nucleotide substitution model (considering the Bayesian Information Criterion, jModelTest 2.1) and a lognormal ucld.mean (mean = 5.0, *SD* = 1.0). Four independent runs were performed from a random starting tree for 80 million generations, with a sampling frequency of 8,000. Resulting log files of the individual runs were imported in Tracer v1.6 (Rambaut, Suchard, Xie, & Drummond, 2014) to check trace plots for convergence (= straight hairy‐caterpillar profile; Drummond, Ho, Rawlence, & Rambaut, 2007) and effective sample size (ESS). ESS values were well above 200 and so a minimum burn‐in of 10% was selected for all three runs. Log files and trees files were combined in LogCombiner v.1.8.4 (Drummond, Suchard, Xie, & Rambaut, 2012) after removal of burn‐in. TreeAnnotator v1.8.4 was used to generate consensus trees (0% burn‐in) and to infer the Maximum Clade Credibility (MCC) tree, presenting the highest product of individual clade posterior probabilities. Final trees with bootstrap values (BS) and posterior probabilities (pp) were visualized in FigTree v1.4.3 (tree.bio.ed.ac.uk/software/figtree/).

### 2.5 Diversity in *Gloeandromyces*

To assess phylogenetic diversity within the genus *Gloeandromyces*, the LSU rDNA dataset was used. This region was put forward by previous studies to replace ITS as barcode for species delimitation in Laboulbeniomycetes (D. Haelewaters et al., unpublished data; Walker et al., 2018). Maximum likelihood (ML) analysis was run using the PAUP on XSEDE 4.0b tool (Swofford, 1991). The appropriate nucleotide substitution model was selected statistically with the help of jModelTest 2.1 (Darriba et al., 2012) by considering the Akaike Information Criterion (AIC). A transitional substation model (TIM2) with the assumption of a gamma distribution (+G) gave the best scoring tree (− lnL = 2,114.8480). Rapid bootstrapping (BS) was implemented with 500 replicates. Next, for our Bayesian inference approach, two independent MCMC chains were conducted under a strict molecular clock, with a Yule speciation tree prior (Gernhard, 2008; Yule, 1925) and the TPM2uf+G model of nucleotide substitution as selected by the Bayesian Information Criterion from jModelTest 2.1. The runs were performed from a random starting tree for 40 million generations, with sampling of parameters and trees every 4,000 generations. The two resulting log files were combined in LogCombiner v1.8.4 with 10% burn‐in. Consensus trees with 0% burn‐in were generated and the MCC tree was constructed in TreeAnnotator v.1.8.4.

### 2.6 Comparison of host and Laboulbeniales phylogenies

Sequence data for analyses were obtained by taking a single isolate per species for both the hosts and Laboulbeniales. For bat flies, mitochondrial cytochrome oxidase gene subunit I (COI) sequences were used. The bat fly dataset included: *Brachytarsina alluaudi* (outgroup); *Exastinion clovisi*,* Megistopoda aranea*,* Nycteribia schmidlii*,* Penicillidia conspicua*,* P. monoceros*,* Speiseria ambigua*,* Trichobius costalimai*,* Tri. dugesioides*,* Tri. joblingi*,* Tri. parasiticus*,* Tri. yunkeri* (hosts); *Mastoptera guimaraesi*,* Paratrichobius longicrus*,* Strebla wiedemanni* (to add structure and provide support to the tree). *Penicillidia monoceros* is not a host to Laboulbeniales, but this bat fly species was selected as a substitute for *P. dufourii*, for which no sequences exist. For Laboulbeniales, large subunit ribosomal DNA (LSU rDNA) sequences were used. The dataset of Laboulbeniales included the following species: *Herpomyces periplanetae* (outgroup); *Arthrorhynchus nycteribiae*,* G. nycteribiidarum*,* G. pageanus*,* G*. spp. nov. 1–4, *G. streblae* Clade A, *G. streblae* Clade B, *Nycteromyces streblidinus* (species associated with bat flies); *Hesperomyces virescens*,* Polyandromyces coptosomalis*,* Stigmatomyces protrudens* (to add structure and provide support to the tree). Sequences were aligned in Muscle v3.7 (Edgar, 2004) on Cipres. Alignments were visually inspected in BioEdit v7.2.6 (Hall, 1999). Maximum likelihood (ML) phylogenetic trees were generated using RAxML v8.2.X (Stamatakis, 2014) available on Cipres. ML was inferred under a GTRCAT model, with 1,000 bootstrapping replicates. To visualize host–Laboulbeniales interactions, cladograms were generated from the best ML trees in FigTree v1.4.3 and saved as NEXUS files. The co‐phylogeny plot was constructed in R (R Core Team, 2013) using the package “ape” (Paradis, Claude, & Strimmer, 2004).

### 2.7 Associations network

All presence/absence data of Laboulbeniales on bat flies and bat flies on bats were entered in a database. Data were partitioned to represent distinct climatic zones (temperate, neotropical). The bat–bat fly–Laboulbeniales associations were visualized with the help of the R package “bipartite” (Dormann, Gruber, & Fründ, 2008). Weighted data and the function plotweb were used to build a network showing host‐dependencies and prevalence. Bats and bat flies that were not identified to genus level, bats without specimen label and infected bat flies with unidentified Laboulbeniales were excluded from the analysis. Bats and bat flies for which *n* < 10 were also excluded.

## 3 RESULTS

### 3.1 Nucleotide alignment datasets

We generated 54 sequences of bat fly‐associated Laboulbeniales during this study, of which 26 SSU and 28 LSU sequences. Our SSU + LSU concatenated dataset comprised 3,969 characters, of which 2,962 were constant and 789 were parsimony‐informative. A total of 84 isolates were included (Table 1): *Arthrorhynchus* (2), *Camptomyces* (1), *Fanniomyces* (1), *Gloeandromyces* (26), *Herpomyces* (7, outgroup), *Hesperomyces* (22), *Nycteromyces* (5), *Polyandromyces* (2), *Prolixandromyces* (1), *Rickia* (4) and *Stigmatomyces* (13). Our LSU dataset consisted of 27 isolates (including 1 *Stigmatomyces* as outgroup) and 955 characters, of which 817 were constant and 110 were parsimony‐informative.

### 3.2 Phylogenetic inferences

**Figure 3:**
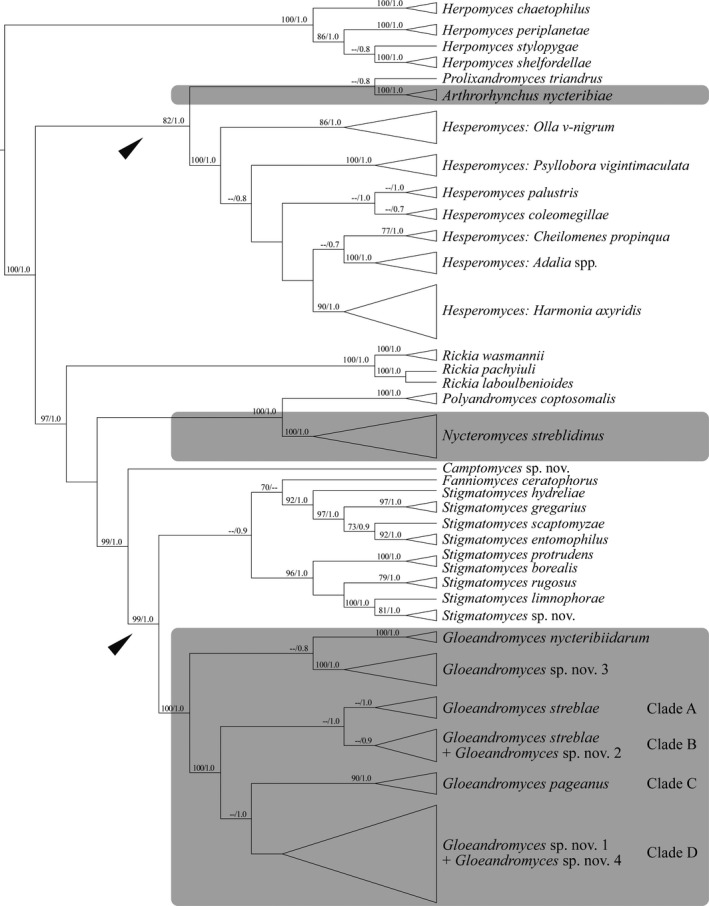
Maximum clade credibility tree, reconstructed from the concatenated SSU + LSU dataset. The tree is the result of a Bayesian analysis performed in BEAST. For each node, ML BS (if ≥70)/Bayesian pp (if ≥0.7) are presented above to the branch leading to that node. The arrowheads denote the Stigmatomycetinae subtribe *sensu* Tavares (1985)

The three genera of bat fly‐associated Laboulbeniales occur in three disparate places of our phylogenetic reconstruction of the SSU + LSU dataset (Figure 3): *Arthrorhynchus nycteribiae* is placed in a sister relationship to *Prolixandromyces triandrus* with pp = 0.8; *Nycteromyces streblidinus* is placed in a sister relationship to *Polyandromyces coptosomalis* with maximum support; and the genus *Gloeandromyces* is placed sister to the genus *Stigmatomyces*, with very strong support (ML BS = 99, pp = 1.0). The subtribe Stigmatomycetinae, which holds several genera included in our dataset (Table 2), is a polyphyletic taxon.

**Table 2:** Genera included in the concatenated SSU + LSU dataset, with classification up to ordinal level

Order	Genus	Subtribus	Tribus	Subfamily
Herpomycetales	*Herpomyces*		Herpomycetaceae	
Laboulbeniales	*Arthrorhynchus*	Stigmatomycetinae	Laboulbenieae	Laboulbenioideae
Laboulbeniales	*Camptomyces*	Haplomycetinae	Haplomyceteae	Peyritschielloideae
Laboulbeniales	*Fanniomyces*	Stigmatomycetinae	Laboulbenieae	Laboulbenioideae
Laboulbeniales	*Gloeandromyces*	Stigmatomycetinae	Laboulbenieae	Laboulbenioideae
Laboulbeniales	*Hesperomyces*	Stigmatomycetinae	Laboulbenieae	Laboulbenioideae
Laboulbeniales	*Nycteromyces*	N/A	Dimorphomyceteae	Peyritschielloideae
Laboulbeniales	*Polyandromyces*	N/A	Dimorphomyceteae	Peyritschielloideae
Laboulbeniales	*Prolixandromyces*	Stigmatomycetinae	Laboulbenieae	Laboulbenioideae
Laboulbeniales	*Rickia*	Peyritschiellinae	Peyritschielleae	Peyritschielloideae
Laboulbeniales	*Stigmatomyces*	Stigmatomycetinae	Laboulbenieae	Laboulbenioideae

**Figure 4:**
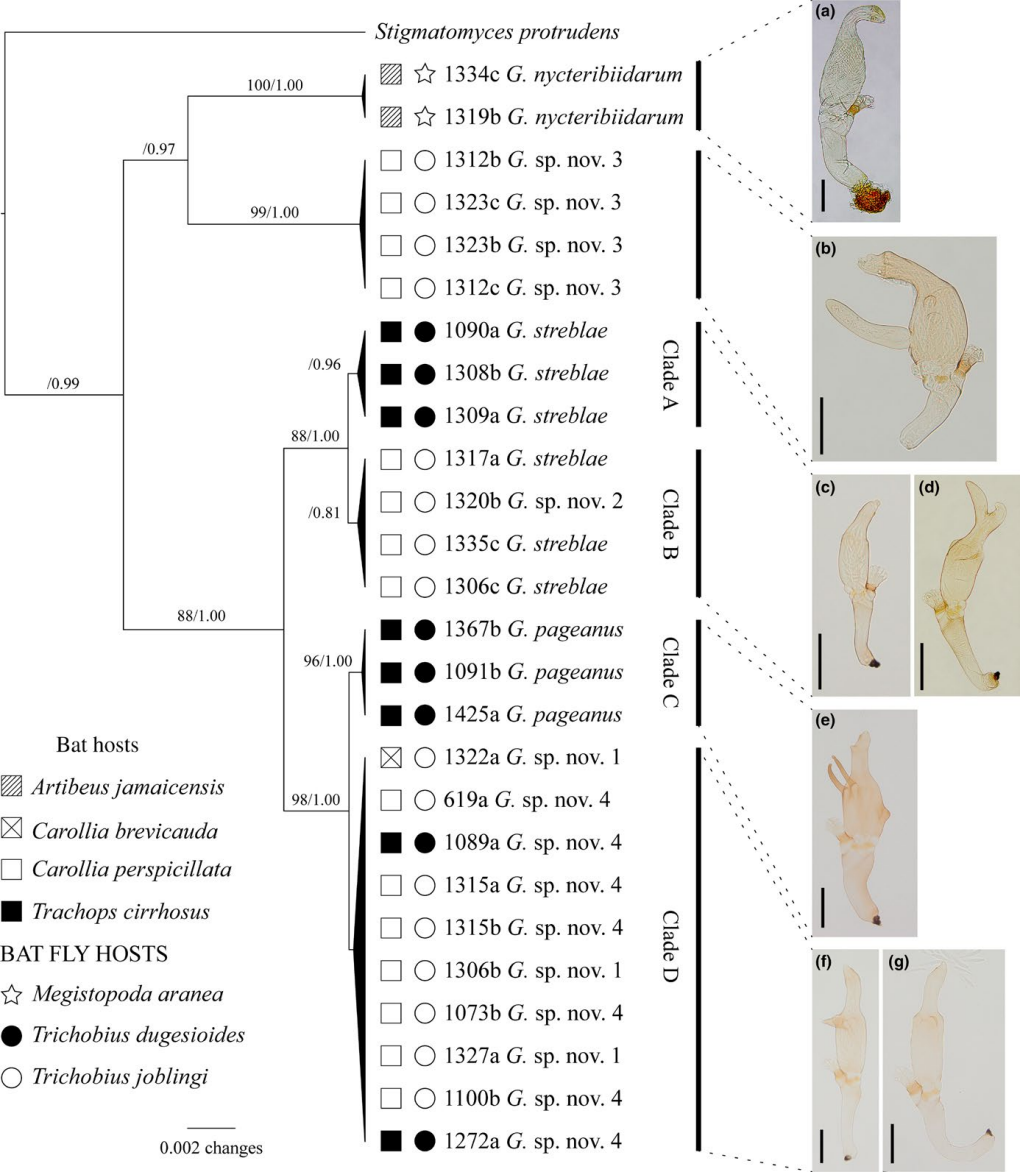
Maximum clade credibility tree showing species in the genus *Gloeandromyces*, with *Stigmatomyces protrudens* as outgroup. The tree is the result of a Bayesian analysis of the LSU dataset performed in BEAST. For each node, ML BS (if ≥70)/Bayesian pp (if ≥0.7) are presented above the branch leading to that node. At the right, thalli are shown of the different morphologies observed. From top to bottom: *Gloeandromyces nycteribiidarum*,* G*. sp. nov. 3, *G. streblae* (left) and *G*. sp. nov. 2 (right), *G. pageanus*,* G*. sp. nov. 1 (left), and *G*. sp. nov. 4 (right)

In the LSU dataset, *Gloeandromyces* forms six distinct clades (Figure 4). *Gloeandromyces nycteribiidarum* and *G*. sp. nov. 3 (*sensu* Walker et al., 2018) are sister taxa and have high support. *Gloeandromyces streblae* falls apart into two clades A and B, each lacking ML BS support but with moderate to high pp support. *Gloeandromyces* sp. nov. 2 (*sensu* Walker et al., 2018) falls in Clade B, among isolates of *G. streblae*. Clade C includes isolates of the recently described *G. pageanus*. Support for clade C is high (BS = 96, pp = 1.00) whereas support is lacking for its sister clade D, which includes isolates of *G*. sp. nov. 1 (*sensu* Walker et al., 2018) and another undescribed form, *Gloeandromyces* sp. nov. 4. All isolates included in clade D are identical in their LSU. Out of the 955 nucleotides, three are different between the isolates in clade C and those in clade D.

### 3.3 Bats, bat flies and Laboulbeniales

**Figure 5:**
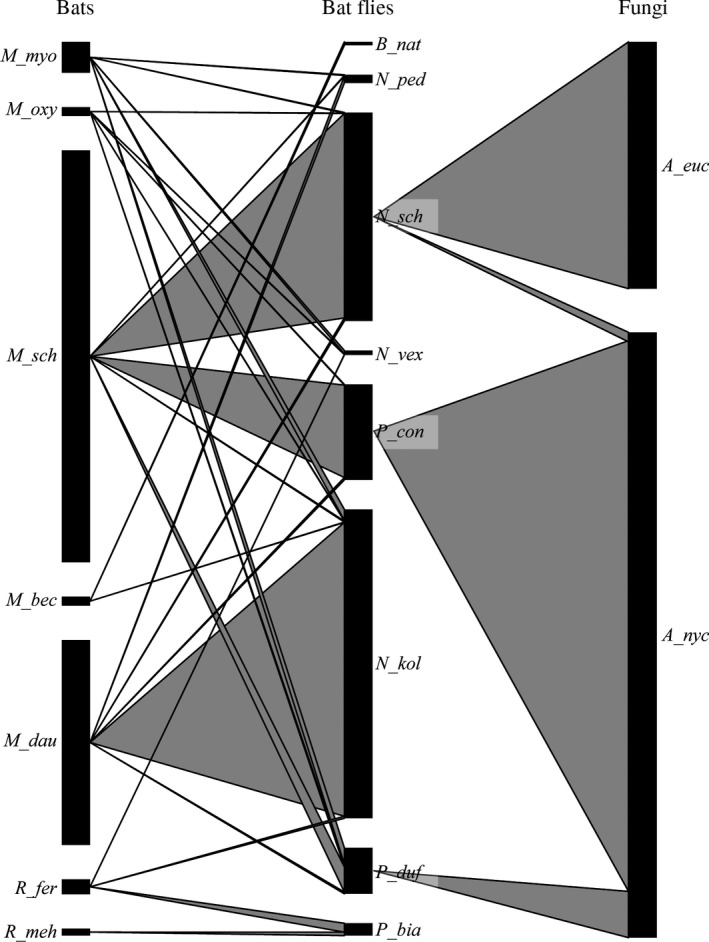
Host–parasite–parasite network of the final temperate dataset. Shown is the association of bat flies with their bat hosts (*left*) as well as the association of Laboulbeniales (*right*) and their bat fly hosts. Bar width represents the relative abundance of a species within each network level

Our complete dataset, prior to excluding specimens and partitioning (Supporting Information), was composed of 2,599 bats and 7,949 bat flies, of which 363 (= 4.6%) were infected by Laboulbeniales. Seven bat species were included in our final temperate dataset (Haelewaters et al., 2017a; Szentiványi et al., 2018). The most abundantly parasitized bat species was *Miniopterus schreibersii* (*n* = 414), followed by *Myotis daubentonii* (*n* = 206). Eight species of bat flies were removed from bats: *Basilia natali* (*n* = 10), *Nycteribia kolenatii* (*n* = 899), *N. pedicularia* (*n* = 24), *N. schmidlii* (*n* = 607), *N. vexata* (*n* = 13), *Penicillidia conspicua* (*n* = 278), *P. dufourii* (*n* = 134) and *Phthiridium biarticulatum* (*n* = 36). The highest number of bat flies was found on *M. schreibersii* bats (*n* = 942 bat flies altogether), closely followed by *M. daubentonii* (*n* = 896 bat flies). On the other bat species, less than 100 bat flies per species were found altogether. Laboulbeniales infection was found on three bat fly species: *Nycteribia schmidlii* (*n* = 26 + 1), *Penicillidia conspicua* (*n* = 59), and *P. dufourii* (*n* = 6). The overall parasite prevalence of Laboulbeniales on temperate bat flies was 4.6%. *Nycteribia schmidlii* was host for two species of Laboulbeniales, *A. eucampsipodae* (*n* = 26) and *A. nycteribiae* (*n* = 1). Both *Penicillidia* host species only carried *A. nycteribiae* thalli. Associations are shown in Figure 5.

**Figure 6:**
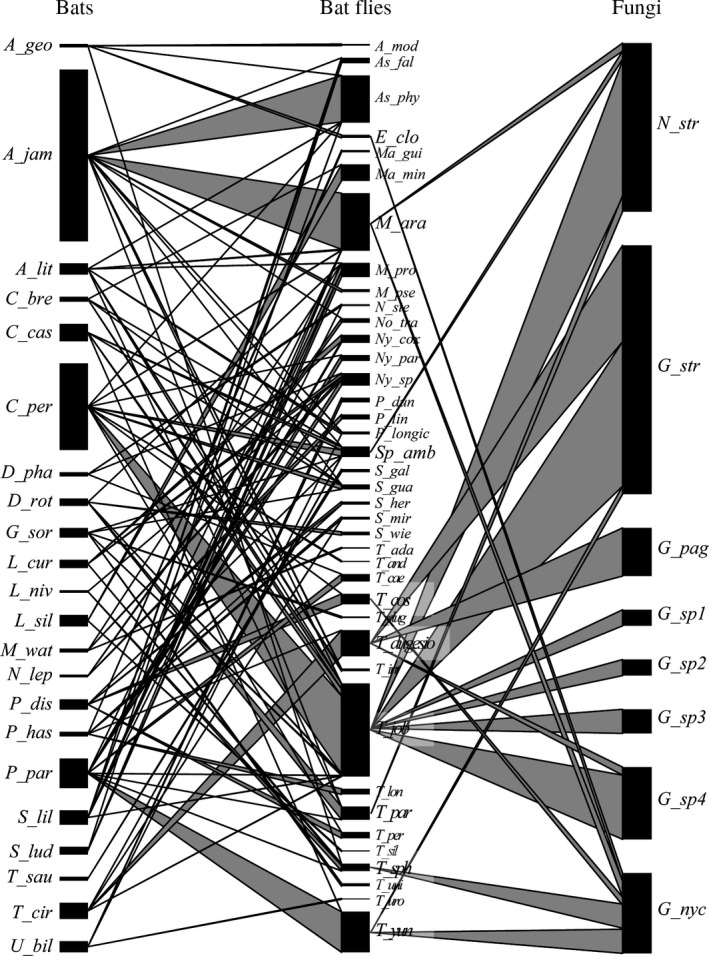
Host–parasite–parasite network of the final neotropical dataset. Shown is the association of bat flies with their bat hosts (*left*) as well as the association of Laboulbeniales (*right*) and their bat fly hosts. Bar width represents the relative abundance of a species within each network level

In our neotropical dataset (Figure 6) 1,703 bats were present, *Artibeus jamaicensis* (*n* = 660), *Carollia perspicillata* (*n* = 333) and *Pteronotus parnellii* (*n* = 114) being the most abundant in addition to 19 other species (with each <70 individuals). The highest number of bat flies was found on *A. jamaicensis* bats (*n* = 1,309 bat flies altogether), followed by *C. perspicillata* (*n* = 1,102), *P. parnellii* (*n* = 755), and *Trachops cirrhosus* (*n* = 334). Of 39 sampled species of bat fly species, nine carried Laboulbeniales thalli (in decreasing order): *Trichobius joblingi* (*n* = 50 infected specimens), *Tri. dugesioides* (*n* = 19), *Tri. yunkeri* (*n* = 4), *Megistopoda aranea*,* Tri. sphaeronotus* (*n* = 3), *Tri. parasiticus* (*n* = 2), *Exastinion clovisi*,* Speiseria ambigua*, and *Tri. costalimai* (*n* = 1). The most frequently encountered species of Laboulbeniales was *Gloeandromyces streblae* (on 33 bat flies of three species), followed by *Nycteromyces streblidinus* (on 21 bat flies of four species). *Trichobius joblingi* was not only most often infected with Laboulbeniales, it also bore the highest number of Laboulbeniales taxa: *Gloeandromyces* sp. nov. 1, *G*. sp. nov. 2, *G*. sp. nov. 3, *G*. sp. nov. 4, *G. streblae*, and *N. streblidinus*. *Gloeandromyces nycteribiidarum* had the highest number of host species: *E. clovisi*,* Megistopoda aranea*,* Tri. costalimai*,* Tri. sphaeronotus* and *Tri. yunkeri*.

### 3.4 Co‐phylogenetic relationships between bat flies and Laboulbeniales

**Figure 7:**
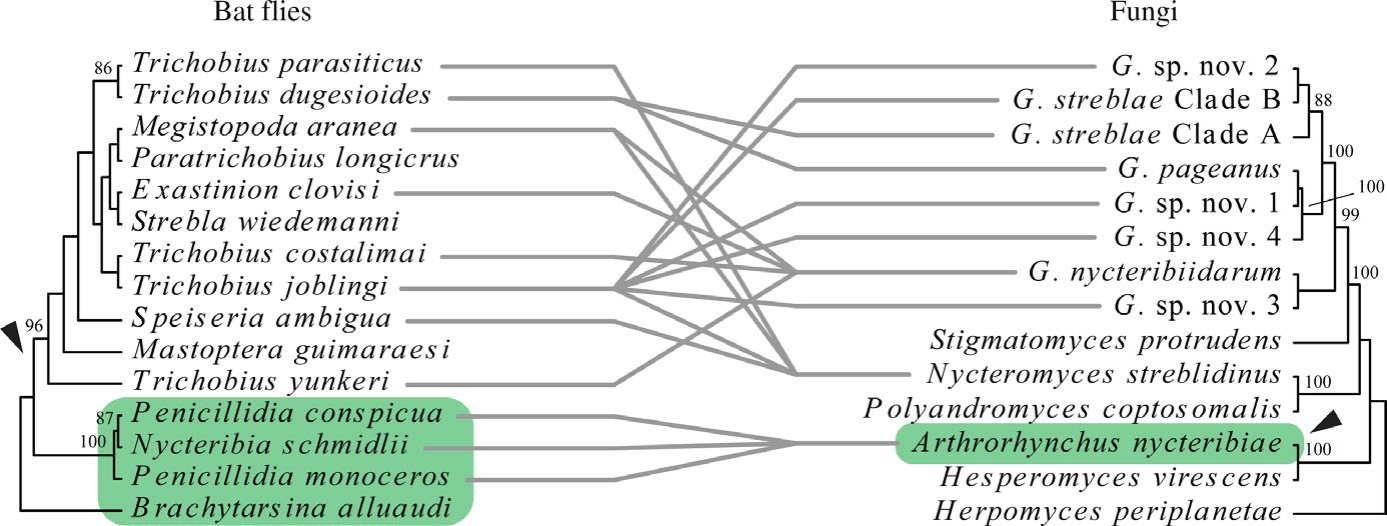
Co‐phylogenetic relationships between bat flies and Laboulbeniales. Maximum likelihood phylogenies for bat flies (left) and their Laboulbeniales parasites (right). For each node, ML BS (if ≥70) are presented above the branch leading to that node. All associations are shown as gray connecting lines. Old World bat flies and Laboulbeniales are highlighted in green. *Penicillidia monoceros* substituted for *Penicillidia dufourii*

Our COI dataset of bat flies consisted of 15 taxa (one outgroup) and 677 characters, of which 410 were constant and 177 were parsimony‐informative. Our LSU dataset of Laboulbeniales consisted of 14 taxa (1 outgroup) and 998 characters, of which 610 were constant and 217 were parsimony‐informative. The co‐phylogeny plot is shown in Figure 7. There is congruence between the (basal‐most) Old World clades, otherwise the evidence for coevolution is lacking.

## 4 DISCUSSION

### 4.1 Bats and bat flies in Panama

Bats are the most diverse mammal group in Panama, with a total of 119 documented species (Moras, Gregorin, Sattler, & Tavares, 2018; Samudio & Pino, 2014). Although species reports are numerous, many come from lowland research (Handley, 1966; Samudio, 2002). This implies that mammal inventories have not been conducted in many highland Panamanian regions, such as Chiriquí and the unexplored Darién Gap. We chose to conduct intensive fieldwork in one such area, a private cloud‐forested nature reserve in Darién, Reserva Natural Chucantí, managed by the NGO Adopt a Panama Rainforest (ADOPTA). Most of the bat flies infected by species of Laboulbeniales used in this study were collected in this reserve. With a team of six, we captured bats at Chucantí for seven nights, investing 68 mnh (mistnet hours, 1 mnh = a single 6 m‐wide mistnet open for 1 hr). We captured 227 bats representing 17 species. We captured *Micronycteris schmidtorum*, a species reported previously only from the Los Santos Province (Handley, 1966). In addition, we encountered the rarely collected *Platyrrhinus dorsalis*, representing the westernmost report of this species (Velazco, 2005). Of the captured bats, 148 carried bat flies (65%). The number of sampled bat flies was 437, representing 16 species. One species was a new country record (*Trichobius anducei*) and five species represented first reports for Darién (*Basilia anceps*,* Anatrichobius scorzai*,* Nycterophilia parnelli*,* Tri. johnsonae*,* Tri. parasiticus*) (Guerrero, 1998a; Lourenço, Almeida, & Famadas, 2016; Stamper, 2012; Table 3). Of all screened bat flies, 30 bore species of Laboulbeniales (6.86%). The results of the tripartite survey at Chucantí were published by Walker et al. (2018).

**Table 3:** All species of bat flies reported in Panama to date. Bat flies reported as host to Laboulbeniales fungi are bolded, details are provided in the last column

Bat fly species	Reference(s)	Reported Laboulbeniales taxa
Nycteribiidae		
*Basilia anceps*	Guimarães (1966), Walker et al. (2018)	
*Basilia dunni*	Guimarães (1966)	
*Basilia ferruginea*	Guimarães (1966)	
*Basilia handleyi*	Guimarães (1966)	
*Basilia myotis*	Guimarães (1966)	
*Basilia tiptonii*	Guimarães (1966)	
*Basilia wenzeli*	Guimarães (1966)	
Streblidae		
*Anastrebla mattadeni*	Wenzel and Tipton (1966)	
*Anastrebla modestini*	Wenzel and Tipton (1966)	
*Anastrebla nycteridis*	Wenzel and Tipton (1966)	
*Anatrichobius scorzai*	Wenzel and Tipton (1966), Walker et al. (2018)	
*Aspidoptera phyllostomatis*	González et al. (2004), Wenzel and Tipton (1966), Walker et al. (2018)	
*Aspidoptera delatorrei*	González et al. (2004), Wenzel and Tipton (1966)	
*Eldunnia breviceps*	Wenzel and Tipton (1966)	
***Exastinion clovisi***	Wenzel and Tipton (1966)	*Gloeandromyces nycteribiidarum*, Mexico (this study); Laboulbeniales gen. & sp. indet., Brazil (Bertola et al., 2005)
*Joblingia schmidti*	Wenzel and Tipton (1966)	
*Mastoptera guimaraesi*	González et al. (2004), Wenzel and Tipton (1966)	
*Mastoptera minuta*	Wenzel and Tipton (1966)	
***Megistopoda aranea***	González et al. (2004), Wenzel and Tipton (1966), Walker et al. (2018)	*Gloeandromyces nycteribiidarum*, Grenada (Thaxter, 1917), Panama (Walker et al., 2018); *G. streblae* & *Nycteromyces streblidinus*, Panama (Walker et al., 2018); Laboulbeniales gen. & sp. indet., Brazil (Bertola et al., 2005)
***Megistopoda proxima***	Wenzel and Tipton (1966), Walker et al. (2018)	Laboulbeniales gen. & sp. indet., Brazil (Bertola et al., 2005)
*Megistopoda theodori*	Wenzel and Tipton (1966)	
*Metelasmus pseudopterus*	González et al. (2004), Wenzel and Tipton (1966)	
*Neotrichobius stenopterus*	González et al. (2004), Wenzel and Tipton (1966)	
*Noctiliostrebla maai*	Wenzel and Tipton (1966)	
*Noctiliostrebla traubi*	Wenzel and Tipton (1966)	
*Nycterophilia fairchildi*	Wenzel and Tipton (1966)	
*Nycterophilia natali*	Wenzel and Tipton (1966)	
*Nycterophilia parnelli*	Wenzel and Tipton (1966), Walker et al. (2018)	
*Paradyschiria lineata*	Wenzel and Tipton (1966)	
*Paradyschiria parvuloides*	Wenzel and Tipton (1966)	
*Parastrebla handleyi*	Wenzel and Tipton (1966)	
*Paratrichobius dunni*	González et al. (2004), Wenzel and Tipton (1966)	
***Paratrichobius longicrus***	Wenzel and Tipton (1966), Walker et al. (2018)	Laboulbeniales gen. & sp. indet., Brazil (Bertola et al., 2005)
*Paratrichobius lowei*	Wenzel and Tipton (1966)	
*Paratrichobius salvini*	González et al. (2004), Wenzel and Tipton (1966)	
*Paratrichobius sanchezi*	Wenzel and Tipton (1966)	
*Paratrichobius* sp. (*longicrus* complex)	Wenzel and Tipton (1966)	
*Pseudostrebla greenwelli*	Wenzel and Tipton (1966)	
*Pseudostrebla ribeiroi*	Wenzel and Tipton (1966)	
***Speiseria ambigua***	González et al. (2004), Wenzel and Tipton (1966), Walker et al. (2018)	*Gloeandromyces streblae*, Ecuador; *Nycteromyces streblidinus*, Honduras (this study); Laboulbeniales gen. & sp. indet., Costa Rica (Fritz, 1983)
*Strebla altmani*	Wenzel and Tipton (1966)	
*Strebla alvarezi*	Wenzel and Tipton (1966)	
***Strebla guajiro***	González et al. (2004), Wenzel and Tipton (1966), Walker et al. (2018)	Laboulbeniales gen. & sp. indet., Costa Rica (Fritz, 1983)
*Strebla christinae*	Wenzel and Tipton (1966)	
*Strebla diaemi*	Wenzel and Tipton (1966)	
*Strebla galindoi*	Wenzel and Tipton (1966)	
*Strebla hertigi*	Wenzel and Tipton (1966)	
*Strebla hoogstraali*	Wenzel and Tipton (1966)	
*Strebla kohlsi*	Wenzel and Tipton (1966)	
*Strebla mirabilis*	González et al. (2004), Wenzel and Tipton (1966)	
***Strebla wiedemanni***	Wenzel and Tipton (1966)	*Gloeandromyces streblae* & *Nycteromyces streblidinus*, Venezuela (Thaxter, 1917)
*Trichobioides perspicillatus*	Wenzel and Tipton (1966)	
*Trichobius anducei*	Walker et al. (2018)	
*Trichobius bequarti*	Wenzel and Tipton (1966)	
*Trichobius brennani*	Wenzel and Tipton (1966)	
***Trichobius costalimai***	Wenzel and Tipton (1966)	*Gloeandromyces nycteribiidarum*, Panama (this study)
***Trichobius dugesii***	Wenzel and Tipton (1966)	Laboulbeniales gen. & sp. indet., Brazil (Bertola et al., 2005)
***Trichobius dugesioides***	Wenzel and Tipton (1966), Walker et al. (2018)	*Gloeandromyces pageanus* & *G. streblae*, Panama (Haelewaters et al., 2017b; Walker et al., 2018)
*Trichobius dunni*	Wenzel and Tipton (1966)	
*Trichobius galei*	Wenzel and Tipton (1966)	
***Trichobius joblingi***	González et al. (2004), Wenzel and Tipton (1966), Walker et al. (2018)	*Gloeandromyces* spp. nov. 1–4 & *G. streblae*, Panama (Haelewaters et al., 2017b; Walker et al., 2018); Laboulbeniales gen. & sp. indet., Costa Rica (Fritz, 1983)
*Trichobius johnsonae*	Wenzel and Tipton (1966), Walker et al. (2018)	
*Trichobius keenani*	Wenzel and Tipton (1966)	
*Trichobius lionycteridis*	Wenzel and Tipton (1966)	
*Trichobius lonchophyllae*	Wenzel and Tipton (1966)	
*Trichobius longipes*	González et al. (2004), Wenzel and Tipton (1966)	
*Trichobius macrophylli*	Wenzel and Tipton (1966)	
*Trichobius mendezi*	Wenzel and Tipton (1966)	
***Trichobius parasiticus***	Wenzel and Tipton (1966), Walker et al. (2018)	*Nycteromyces streblidinus*, Honduras (this study)
*Trichobius sparsus*	González et al. (2004), Wenzel and Tipton (1966)	
***Trichobius uniformis***	Wenzel and Tipton (1966)	Laboulbeniales gen. & sp. indet., Brazil (Bertola et al., 2005)
*Trichobius urodermae*	Wenzel and Tipton (1966)	
*Trichobius vampyropis*	Wenzel and Tipton (1966)	
***Trichobius yunkeri***	Wenzel and Tipton (1966), Walker et al. (2018)	*Gloeandromyces nycteribiidarum*, Costa Rica; *G. streblae*, Panama (Haelewaters et al., 2017b)

### 4.2 Prevalences

A comprehensive study of nycteribiid bat fly‐associated Laboulbeniales was conducted by Blackwell (1980b). She screened 2,517 bat flies, of which 56 were infected with *Arthrorhynchus eucampsipodae* or *A. nycteribiae*, denoting a parasite prevalence of 2.2%. In our larger study, we screened 7,949 bat flies of which 363 were infected by Laboulbeniales (4.6%). This includes both temperate and neotropical material. Taking only temperate flies into consideration (*n* = 2,001), parasite prevalence was again 4.6%. These low percentages can be explained by life history traits of the bat flies. Deposition of third instar larvae occurs on roosting substrates. Therein lies some risk, because flies need to return to their host within 25 hr. Since the flies are so closely tied to their bat host, we assume that transmission of ascospores of the fungi only occurs on the bat itself, most likely through direct contact (De Kesel, 1995). Host grooming is the main cause of death for bat flies (Marshall, 1981). Apparently, this behavior is an important selective factor driving evolution of host specific and even position‐specific parasites (ter Hofstede, Fenton, & Whitaker, 2004) and may to some extent be an explanatory factor in the observed patterns of Laboulbeniales.

Several studies confirm that bats are often infected by several bat fly species (Dick & Gettinger, 2005; Wenzel, 1976; Wenzel, Tipton, & Kiewlicz, 1966). At the same time, the average number of (nycteribiid) bat flies on their bat hosts is only 1.79 (Haelewaters et al., 2017a). This number depends on bat host species and is much higher for *Myotis daubentonii* (up to 21) and *Miniopterus schreibersii* (up to 13). A majority of Laboulbeniales species are strictly host specific. For those taxa occurring on several host species, such as *Arthrorhynchus nycteribiae*, caution is required in the assessment of their ecology—it is possible that these represent more than a single species. All in all, the number of times an infected bat fly comes into contact with new potential hosts (of the same species) may be very low.

### 4.3 Independent lineages of bat fly‐associated Laboulbeniales

Parasitism of bat flies by Laboulbeniales arose at least three times independently, once in the Eastern Hemisphere and twice in the Western Hemisphere. The genus *Gloeandromyces* is placed sister to the speciose genus *Stigmatomyces*, species of which infect only flies. The other two bat fly‐associated genera form two separate clades, both sisters to a genus of Laboulbeniales that is associated with true bugs (Hemiptera). *Arthrorhynchus* and *Prolixandromyces* form a clade with moderate Bayesian support. The genus *Prolixandromyces* consists of eight species parasitizing taxa in the semi‐aquatic family Veliidae (Weir, 2008). *Nycteromyces* forms a clade with *Polyandromyces*; the basal node of this clade received maximum support. *Polyandromyces* is a monotypic genus; its sole representative, *P. coptosomalis*, occurs on terrestrial species in the families Pentatomidae and Plataspidae. In other words, using the phylogenetic reconstruction of the SSU + LSU dataset, for the first time including molecular data from the rarely sampled bat fly‐associated Laboulbeniales, we identified two interordinal host shifts (true bugs to bat flies). We hypothesize that two bat fly‐associated lineages, *Arthrorhynchus* and *Nycteromyces*, have independently evolved from lineages of true bug ectoparasites. Tavares (1985) noted that bugs are secondary hosts to Laboulbeniales, and that their fungus parasites arose from taxa occurring on beetles (Coleoptera). We cannot confirm this suggestion because our phylogenetic reconstruction is far from complete and does not encompass many taxa with beetle hosts. However, it is clear that Laboulbeniales on beetle hosts are evolutionary very successful; 80% of known species are reported from beetles (Weir & Hammond, 1997). In contrast, the numbers of known species from bugs is 4%, whereas the number from bat flies is less than 1%.

Is it possible bat fly‐associated lineages have evolved from bug‐associated lineages? Representatives of both host groups make use of the bat microhabitat and roost environment. Two families of terrestrial bugs are known as obligatory hematophagous ectoparasites: Cimicidae and Polyctenidae (Schuh & S̆tys, 1991). Both families belong to the superfamily Cimicoidea, along with Anthocoridae, Lasiochilidae, Lyctocoridae, and Plokiophilidae (Jung, Kim, Yamada, & Lee, 2010; Schuh & S̆tys, 1991). One lasiochilid, *Lasiochilus pallidulus*, has been found as a host to *Cupulomyces lasiochili* in Grenada, a member of the Stigmatomycetinae subtribe (Benjamin, 1992a). Benjamin (1992a) used the family name Anthocoridae for the host but he probably used this in the broad sense, whereas Schuh and S̆tys (1991) proposed to split up this non‐monophyletic family into three, Anthocoridae sensu stricto, Lasiochilidae, and Lyctocoridae. Lasiochilids live on the ground, under bark and in vegetation (Schuh & Slater, 1995). It is probable that transmission of ascospores occurs now and then between bugs and bat flies and that this at some point in time may have led to segregation of populations, microevolutionary changes and ultimately speciation.

**Figure 8:**
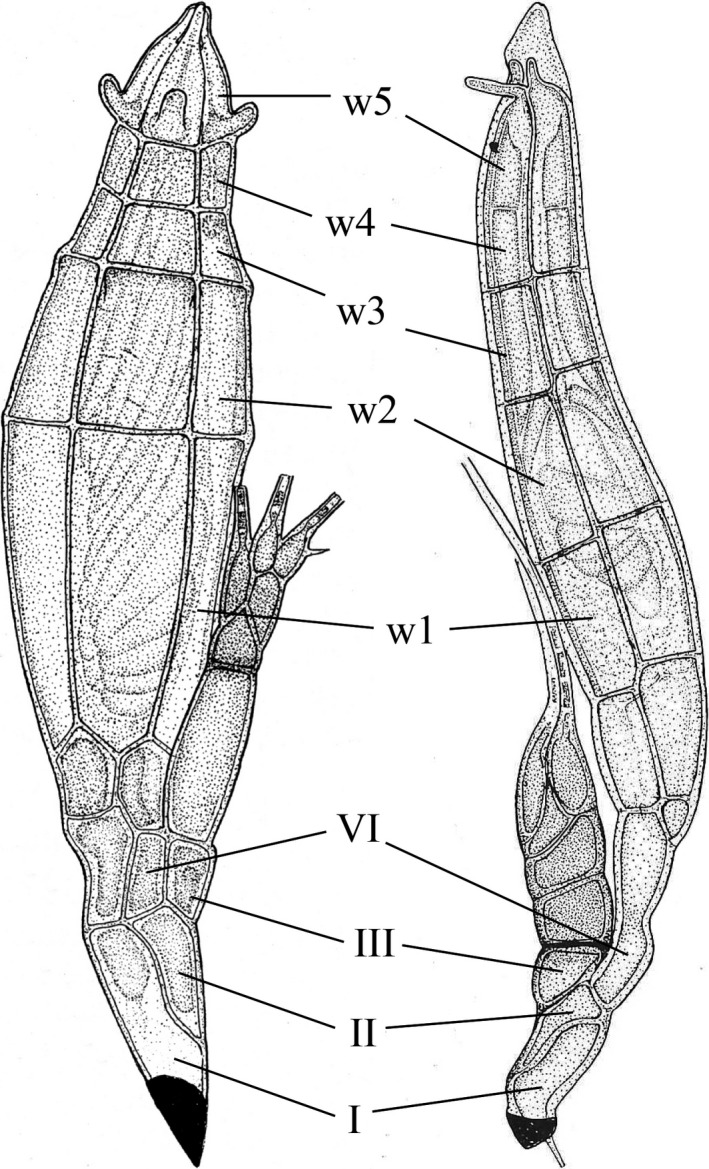
Comparison of two species of Laboulbeniales. Left. Mature thallus of *Cupulomyces lasiochili*, reproduced from Benjamin (1992a). Right. Mature thallus of *Prolixandromyces rhinoceralis*, reproduced from Benjamin (1981). Annotated are cells I, II, III, and VI, and tiers of perithecial outer wall cells (w^1^ to w^5^)

Only *C. lasiochili* has been found on either cimicid or polyctenid bugs, but the limitation with Laboulbeniales reports is that the absence of reports on certain host groups is due to a lack of sampling and screening efforts. We recommend that future studies focus on screening bugs for Laboulbeniales parasites and on generating molecular data for taxa found on bugs. The phylogenetic placement of these taxa, including *C. lasiochili*, will be a crucial data point in evaluating our hypothesis. *Cupulomyces* and *Prolixandromyces*, which is represented in our phylogeny by *P. triandrus*, have a similar receptacle structure (Figure 8): cell II is positioned posterior and next to cell I, separated by an oblique septum, and cell II carries cells III obliquely and VI distally (Benjamin, 1981, 1992a). In *Cupulomyces*, the perithecial wall cells are arranged in five tiers (Benjamin, 1992a). The situation has been described differently for *Prolixandromyces*, where in each vertical row of outer wall cells there are four tiers. However, Tavares (1985) mentioned that the fourth tier “may divide by maturity” even though the septa are extremely thin. Five tiers can be observed in drawings of mature thalli by Benjamin (1981: figure 13, reproduced here) and Weir (2008: figure 10). Consequently, also the perithecial outer wall structure is similar between both genera. Incorporating sequence data for *Cupulomyces* into our phylogenetic reconstruction will help elucidate whether contact between insects in the bat roost environment may have mediated host jumps to and subsequent speciation of Laboulbeniales on bat flies.

### 4.4 Polyphyly of subtribe Stigmatomycetinae

The subtribe Stigmatomycetinae is characterized by a simple receptacle consisting of three superposed cells, of which cell II carries the stalk cell of the perithecium (cell VI) and cell III carries the appendage. Taking synonymies and recent additions into consideration, Stigmatomycetinae now holds 40 genera (Benjamin, 1992a, 1992b, 2001; Santamaria, 1995; Tavares, 1985; Tavares & Balazuc, 1989). Our phylogenetic analysis shows that this subtribe is polyphyletic. We found two well‐supported clades. One clade consists of *Gloeandromyces* and *Stigmatomyces* (including its synonym, *Fanniomyces*), the second clade includes *Arthrorhynchus*,* Hesperomyces*, and *Prolixandromyces*. Even Thaxter's (1908) original circumscription of what he called the “Stigmatomyceteae” tribe, including only five genera, *Acallomyces*,* Acompsomyces*,* Arthrorhynchus*,* Polyascomyces*, and *Stigmatomyces*, is polyphyletic. These findings undermine classification systems of both Thaxter (1908) and Tavares (1985) and are in line with Goldmann and Weir (2018), who retrieved 12 genera of Stigmatomycetinae in three unrelated clades.

### 4.5 Associations between bat flies and Laboulbeniales

Both the temperate bat flies and Laboulbeniales are geographically separated from their neotropical counterparts, so it is no surprise that we observe congruence of the Old World‐clades. The other relationships are difficult to disentangle from an evolutionary point of view. *Nycteromyces streblidinus* is a plurivorous species, with hosts in the genera *Megistopoda*,* Speiseria*, and *Trichobius*. All these are parasitic on phyllostomid bats that commonly roost in hollow trees (Kunz & Lumsdem, 2003; Overal, 1980; Wenzel et al., 1966). Also *G. nycteribiidarum* is plurivorous, with hosts in the genera *Exastinion*,* Megistopoda*, and *Trichobius*. The ecology of the bat hosts of these bat flies is similar. Mormoopidae (*Pteronotus parnellii*, host of *Tri. yunkeri*) almost always roost in caves or mines. *Anoura geoffroyi* (host of *Exastinion clovisi*) and *Phyllostomus discolor* (host of *Tri. costalimai*) preferably roost in caves. The morphospecies within *G. pageanus* and *G. streblae* are restricted to a single host species. We cannot provide an evolutionary explanation for the observed Neotropical patterns in the co‐phylogeny plot, instead we think the patterns can be linked to the roosting ecology of the bat hosts.

*Artibeus* and *Sturnira* are two genera of bats (Phyllostomidae, Stenodermatinae) that use hollow trees as main roosting sites, whereas most other stenodermatine bats roost in foliage or leaf tents (Evelyn & Stiles, 2003; Garbino & Tavares, 2018; Patterson et al., 2007). As a consequence, species of three genera of bat flies parasitize these two host genera. *Megistopoda proxima*,* Metelasmus wenzeli*,* Aspidoptera delatorrei*, and *A. falcata* parasitize species of *Sturnira*; and *Megistopoda aranea*,* Metelasmus pseudopterus* and *Aspidoptera phyllostomatis* parasitize species of *Artibeus* (Graciolli & Dick, 2004). This pattern can be generalized: bats with similar roosting behaviors share similar parasite species. Upon adding another parasite level, it is not hard to imagine that these fungi can be on several, even distantly related species of bat flies, when their bat hosts share the same roosts.

### 4.6 Morphological diversity versus phylogenetic diversity

Based on morphological study, we identified seven species of *Gloeandromyces*. These are *G. nycteribiidarum*,* G. pageanus*,* G. streblae* and four undescribed, putative species (Figure 4a–g). However, this morphological diversity is not reflected in molecular structuring based on the LSU rDNA region. *Gloeandromyces* sp. nov. 1 and *G*. sp. nov. 4 (names consistent with Walker et al., 2018) are identical based on sequence data but show morphological differences. In other words, these isolates are not independent species; instead they represent morphotypes as part of the phenotypic plasticity exhibited by a single phylogenetic species (*sensu* Goldmann & Weir, 2012; Goldmann et al., 2013). In the case of *G. pageanus* and *G. streblae*, we reveal specialization to host species. For *G. streblae*, no obvious morphological features are observed to distinguish between thalli from *Tri. dugesioides* (Clade A) and *Tri. joblingi* (Clade B). In fact, *G. streblae* exhibits high phenotypic plasticity (West‐Eberhard, 1989). In the case of *G. streblae*, this plasticity makes it hard to make morphologically based identifications. Some thalli are morphologically so similar to *G*. sp. nov. 4 that it is difficult to impossible to separate these taxa without sequence data. We have observed and included in our molecular work a range of *G. streblae* thalli, from short, stout, and curved to elongate, some with conspicuous bumps at the distal end of the perithecial venter. Even so, two clades were retrieved that are only segregated by host species. There is one exception: isolate D. Haelew. 1320b represents *Gloeandromyces* sp. nov. 2, which in reality is a morphotype. This morphotype was removed from the last sternite/tergite. We believe the sigmoid habitus of this morphotype is a consequence of morphological adaptions induced by growing on that specific portion of the insect integument.

## 5 CONCLUSIONS

This study has not only substantially increased our knowledge about bats and their ectoparasitic associates, but also shown the need to include molecular data in Laboulbeniales taxonomy. Several phenomena come into play in the morphological and phylogenetic diversity of these parasites. Phenotypic plasticity and position‐induced morphological adaptations go hand in hand. Position‐induced morphotypes belong to the same phylogenetic species. In *Chitonomyces*, transmission of ascospores during mating between hosts seems to be the mechanism leading to position specific morphotypes (Goldmann & Weir, 2012). For bat fly‐associated Laboulbeniales, it is unclear what is driving morphological divergence within phylogenetic species. Another important contributor to diversity, whether or not ephemeral or incipient (Rosenblum et al., 2012), is host specialization. Segregation by host species is observed for at least two bat fly‐associated species. Concerning studies in diversity and taxonomy of Laboulbeniales, our main recommendation is to always include molecular data. The examples discussed in this study have made it clear that it has become impossible to assess diversity by morphology alone.

## CONFLICT OF INTEREST

The authors declare that they have no conflicts of interest with regard to this article.

## DATA ACCESSIBILITY

The complete dataset of bats, batflies and Laboulbeniales is available as Supporting Information (in Excel format). All sequence data used in this study have been submitted to GenBank under accession numbers MH040307 & MH040533–MH040595. Sequence alignments generated during this study (in NEXUS format), input XML and output log and trees files from Bayesian analyses may be accessed from the figshare online repository with the URL https://doi.org/10.6084/m9.figshare.c.4154336.v1.

## AUTHOR CONTRIBUTIONS

D.H. initiated the project, generated all sequence data, performed molecular phylogenetic analyses, prepared all figures, described the new species and morphotypes, and wrote the manuscript with input from R.A.P. and D.H.P. R.A.P. contributed to fieldwork and facilitated research in Panama. D.H.P. provided expertise at all stages of research.

## References

[ece34359-bib-0001] Arnold, A. E. , & Lutzoni, F. (2007). Diversity and host range of foliar fungal endophytes: Are tropical leaves biodiversity hotspots? Ecology, 88, 541–549. 10.1890/05-1459 17503580

[ece34359-bib-0002] Barquez, R. M. , Perez, S. , Miller, B. , & Diaz, M. (2015). Artibeus lituratus. The IUCN red list of threatened species 2015. Available at http://www.iucnredlist.org/details/2136/0 (accessed 06 October 2017).

[ece34359-bib-0501] Benjamin, R. K. (1971). Introduction and supplement to Roland Thaxter's contribution towards a monograph of the Laboulbeniaceae. Bibliotheca Mycologica, 30, 1–155.

[ece34359-bib-0003] Benjamin, R. K. (1981). Laboulbeniales on semiaquatic Hemiptera. IV. Addenda to Prolixandromyces. Aliso, 10, 1–17. 10.5642/aliso

[ece34359-bib-0004] Benjamin, R. K. (1992a). *Cupulomyces*, a new genus of Laboulbeniales (Ascomycetes) based on *Stigmatomyces lasiochili* . Aliso, 13, 355–364. 10.5642/aliso

[ece34359-bib-0005] Benjamin, R. K. (1992b). A new genus of Laboulbeniales (Ascomycetes) on a species of *Phalacrichus* (Coleoptera: Dryopoidea; Limnichidae), with a note on mirror‐image asymmetry in the order. Aliso, 13, 427–446. 10.5642/aliso

[ece34359-bib-0006] Benjamin, R. K. (2001). *Autophagomycetes*,* Bordea*, and a new genus, *Rossiomyces* (Laboulbeniales). Aliso, 19, 99–136.

[ece34359-bib-0007] Benjamin, R. K. , & Shanor, L. (1952). Sex of host specificity and position specificity of certain species of *Laboulbenia* on *Bembidion picipes* . American Journal of Botany, 39, 125–131. 10.1002/j.1537-2197.1952.tb14255.x

[ece34359-bib-0502] Bertola, P. B. , Aires, C. C. , Favorito, S. E. , Graciolli, G. , Amaku, M. , & Pinto‐da‐Rocha, R. (2005). Bat flies (Diptera: Streblidae, Nycteribiidae) parasitic on bats (Mammalia: Chiroptera) at Parque Estadual da Cantareira, São Paulo, Brazil: Parasitism rates and host‐parasite associations. Memorias do Instituto Oswaldo Cruz, 100, 25–32. 10.1590/S0074-02762005000100005 15867959

[ece34359-bib-0008] Blackwell, M. (1980a). Developmental morphology and taxonomic characters of *Arthrorhynchus nycteribiae* and *A. eucampsipodae* (Laboulbeniomycetes). Mycologia, 72, 159–168. 10.2307/3759428

[ece34359-bib-0009] Blackwell, M. (1980b). Incidence, host specificity, distribution, and morphological variation in *Arthrorhynchus nycteribiae* and *A. eucampsipodae* (Laboulbeniomycetes). Mycologia, 72, 143–158. 10.2307/3759427

[ece34359-bib-0011] Darriba, D. , Taboada, G. L. , Doallo, R. , & Posada, D. (2012). jModelTest 2: More models, new heuristics and parallel computing. Nature Methods, 9, 772 10.1038/nmeth.2109 PMC4594756 22847109

[ece34359-bib-0013] De Kesel, A. (1995). Relative importance of direct and indirect infection in the transmission of *Laboulbenia slackensis* (Ascomycetes, Laboulbeniales). Belgian Journal of Botany, 128, 124–130.

[ece34359-bib-0014] De Kesel, A. (1996). Host specificity and habitat preference of *Laboulbenia slackensis* . Mycologia, 88, 565–573. 10.2307/3761150

[ece34359-bib-0015] De Kesel, A. , & Haelewaters, D. (2014). *Laboulbenia slackensis* and *L. littoralis* sp. nov. (Ascomycota, Laboulbeniales), two sibling species as a result of ecological speciation. Mycologia, 106, 407–414. 10.3852/13-348 24871602

[ece34359-bib-0017] Dick, C. W. (2013). Review of the bat flies of Honduras, Central America (Diptera: Streblidae). Journal of Parasitology Research, 2013, 437696 10.1155/2013/437696 23634295 PMC3619636

[ece34359-bib-0503] Dick, C. W. , & Gettinger, D. (2005). A faunal survey of streblid flies (Diptera: Streblidae) associated with bats in Paraguay. Journal of Parasitology, 91, 1015–1024. 10.1645/GE-536R.1 16419742

[ece34359-bib-0019] Dick, C. W. , & Patterson, B. D. (2007). Against all odds: Explaining high host specificity in dispersal‐prone parasites. International Journal for Parasitology, 37, 871–876. 10.1016/j.ijpara.2007.02.004 17382332

[ece34359-bib-0020] Dick, C. W. , & Patterson, B. D. (2008). An excess of males: Skewed sex ratios in bat flies (Diptera: Streblidae). Evolutionary Ecology, 22, 757–769. 10.1007/s10682-007-9201-9

[ece34359-bib-0021] Dittmar, K. , Porter, M. L. , Murray, S. , & Whiting, M. F. (2006). Molecular phylogenetic analysis of nycteribiid and streblid bat flies (Diptera: Brachycera, Calyptratae): Implications for host associations and phylogeographic origins. Molecular Phylogenetics and Evolution, 38, 155–170. 10.1016/j.ympev.2005.06.008 16087354

[ece34359-bib-0022] Dormann, C. F. , Gruber, B. , & Fründ, J. (2008). Introducing the bipartite package: Analysing ecological networks. R News, 8, 8–11.

[ece34359-bib-0023] Drummond, A. J. , Ho, S. Y. W. , Rawlence, N. , & Rambaut, A. (2007) A rough guide to BEAST 1.4. University Auckland, New Zealand. Available at http://beast.bio.ed.ac.uk (accessed 05 March 2018).

[ece34359-bib-0024] Drummond, A. J. , Suchard, M. A. , Xie, D. , & Rambaut, A. (2012). Bayesian phylogenetics with BEAUti and the BEAST 1.7. Molecular Biology and Evolution, 29, 1969–1973. 10.1093/molbev/mss075 22367748 PMC3408070

[ece34359-bib-0025] Duron, O. , Schneppat, U. E. , Berthomieu, A. , Goodman, S. M. , Droz, B. , Paupy, C. , … Tortosa, P. (2014). Origin, acquisition and diversification of heritable bacterial endosymbionts in louse flies and bat flies. Molecular Ecology, 23, 2105–2117. 10.1111/mec.12704 24612422

[ece34359-bib-0026] Eckhart, L. , Bach, J. , Ban, J. , & Tschachler, E. (2000). Melanin binds reversibly to thermostable DNA polymerase and inhibits its activity. Biochemical and Biophysical Research Communications, 271, 726–730. 10.1006/bbrc.2000.2716 10814530

[ece34359-bib-0027] Edgar, R. C. (2004). MUSCLE: Multiple sequence alignment with high accuracy and high throughput. Nucleic Acids Research, 32, 1792–1797. 10.1093/nar/gkh340 15034147 PMC390337

[ece34359-bib-0028] Evelyn, M. J. , & Stiles, D. A. (2003). Roosting requirements of two frugivorous bats (*Sturnira lilium* and *Arbiteus intermedius*) in fragmented Neotropical forest. Biotropica, 35, 405–418. 10.1111/j.1744-7429.2003.tb00594.x

[ece34359-bib-0504] Fritz, G. N. (1983). Biology and ecology of bat flies (diptera: streblidae) on bats in the genus *Carollia* . Journal of Medical Entomology, 20, 1–10. 10.1093/jmedent/20.1.1 6827567

[ece34359-bib-0031] Garbino, G. S. T. , & Tavares, V. C. (2018). 2018. Roosting ecology of Stenodermatinae bats (Phyllostomidae): Evolution of foliage roosting and correlated phenotypes. Mammal Review, 48, 75–89. 10.1111/mam.12114

[ece34359-bib-0032] Gernhard, T. (2008). The conditioned reconstructed process. Journal of Theoretical Biology, 253, 769–778. 10.1016/j.jtbi.2008.04.005 18538793

[ece34359-bib-0033] Goldmann, L. , & Weir, A. (2012). Position specificity in *Chitonomyces* (Ascomycota, Laboulbeniomycetes) on *Laccophilus* (Coleoptera, Dytiscidae): A molecular approach resolves a century‐old debate. Mycologia, 104, 1143–1158. 10.3852/11-358 22684291

[ece34359-bib-0034] Goldmann, L. , & Weir, A. (2018). Molecular phylogeny of the Laboulbeniomycetes (Ascomycota). Fungal Biology, 122, 87–100. 10.1016/j.funbio.2017.11.004 29458722

[ece34359-bib-0035] Goldmann, L. , Weir, A. , & Rossi, W. (2013). Molecular analysis reveals two new dimorphic species of *Hesperomyces* (Ascomycota, Laboulbeniomycetes) parasitic on the ladybird *Coleomegilla maculata* (Coleoptera, Coccinellidae). Fungal Biology, 117, 807–813. 10.1016/j.funbio.2013.10.004 24295919

[ece34359-bib-0505] González, D. P. , Santos, A. M. , & Miranda, J. R. (2004). Streblidae (Diptera: Pupipara) ectoparásitos de murciélagos, en las tierras bajas del Parque Nacional Darien, provincia de Darién, Panamá. Tecnociencias, 6, 1–12.

[ece34359-bib-0036] Graciolli, G. , & Dick, C. W. (2004). A new species of *Metelasmus* (Diptera: Streblidae: Streblinae) from southern South America. Zootaxa, 509, 1–8. https://doi.org/10.11646/zootaxa.509.1.1

[ece34359-bib-0037] Guerrero, R. (1993). Catalogo de los Streblidae (Diptera: Pupipara) parasitos de murcielagos (Mammalia: Chiroptera) del Nuevo Mundo I. Clave para los géneros y Nycterophiliinae. Acta Biologica Venezuelica, 14, 61–75.

[ece34359-bib-0038] Guerrero, R. (1994a). Catalogo de los Streblidae (Diptera: Pupipara) parasitos de murcielagos (Mammalia: Chiroptera) del Nuevo Mundo II. Los grupos: *pallidus*,* caecus*,* major*,* uniformis* y *longipes* del género *Trichobius* (Gervais, 1844). Acta Biologica Venezuelica, 15, 1–18.

[ece34359-bib-0039] Guerrero, R. (1994b). Catalogo de los Streblidae (Diptera: Pupipara) parasitos de murcielagos (Mammalia: Chiroptera) del Nuevo Mundo IV. Trichobiinae con alas desarrolladas. Boletín de Entomología Venezolana, 9, 161–192.

[ece34359-bib-0040] Guerrero, R. (1995a). Catalogo de los Streblidae (Diptera: Pupipara) parasitos de murcielagos (Mammalia: Chiroptera) del Nuevo Mundo III. Los grupos: *dugesii*,* dunni* y *phyllostomae* del género *Trichobius* (Gervais, 1844). Acta Biologica Venezuelica, 15, 1–27.

[ece34359-bib-0041] Guerrero, R. (1995b). Catalogo de los Streblidae (Diptera: Pupipara) parasitos de murcielagos (Mammalia: Chiroptera) del Nuevo Mundo V. Trichobiinae con alas reducidas o ausentes y miscelaneos. Boletín de Entomología Venezolana, 10, 135–160.

[ece34359-bib-0042] Guerrero, R. (1996). Catálogo de los Streblidae (Diptera: Pupipara) parásitos de murciélagos (Mammalia: Chiroptera) del Nuevo Mundo VI. Streblinae. Acta Biologica Venezuelica, 16, 1–26.

[ece34359-bib-0043] Guerrero, R. (1997). Catálogo de los Streblidae (Diptera: Pupipara) parásitos de murciélagos (Mammalia: Chiroptera) del Nuevo Mundo VII. Lista de especies, hospedadores y países. Acta Biologica Venezuelica, 17, 9–24.

[ece34359-bib-0044] Guerrero, R. (1998a). Notes on Neotropical batflies (Diptera, Streblidae). I. The genus *Trichobius*, with description of two new species and one new subspecies from Venezuela. Acta Parasitologica, 43, 86–93.

[ece34359-bib-0045] Guerrero, R. (1998b). Notes on Neotropical bat flies (Diptera: Streblidae): II. Review of the genus *Xenotrichobius* . Acta Parasitologica, 43, 142–147.

[ece34359-bib-0046] Guerrero, J. A. , Ortega, J. , Gonzalez, D. , Maldonado, J. E. , Lorenzo, C. , & Espinoza, E. (2008). Molecular phylogenetics and taxonomy of the fruit‐eating bats of the genus *Artibeus* (Chiroptera: Phyllostomidae). Avances en el Estudio de los Mamiferos de México. Publicaciones Especiales, 2, 125–146.

[ece34359-bib-0047] Guimarães, L. R. (1966). Nycteribiid batflies from Panama (Diptera: Nycteribiidae) In Wenzel R. L., & Tipton V. J. (Eds.), Ectoparasites of Panama (pp. 393–404). Chicago: Field Museum of Natural History.

[ece34359-bib-0048] Haelewaters, D. , Gorczak, M. , Pfliegler, W. P. , Tartally, A. , Tischer, M. , Wrzosek, M. , & Pfister, D. H. (2015). Bringing Laboulbeniales into the 21st century: Enhanced techniques for extraction and PCR amplification of DNA from minute ectoparasitic fungi. IMA Fungus, 6, 363–372. 10.5598/imafungus.2015.06.02.08 26734547 PMC4681260

[ece34359-bib-0049] Haelewaters, D. , Pfliegler, W. P. , Gorczak, M. , & Pfister, D. H. (in review). Birth of an order: Comprehensive phylogenetic study excludes *Herpomyces* (Fungi, Laboulbeniomycetes) from Laboulbeniales. Molecular Phylogenetics and Evolution. 10.1016/j.ympev.2019.01.007 30625361

[ece34359-bib-0050] Haelewaters, D. , Pfliegler, W. P. , Szentiványi, T. , Földvári, M. , Sándor, A. D. , Barti, L. , … Pfister, D. H. (2017a). Parasites of parasites of bats: Laboulbeniales (Fungi: Ascomycota) on bat flies (Diptera: Nycteribiidae) in Central Europe. Parasites & Vectors, 10, 96 10.1186/s13071-017-2022-y 28222795 PMC5320862

[ece34359-bib-0051] Haelewaters, D. , Verhaeghen, S. J. C. , Rios Gonzalez, T. A. , Bernal Vega, J. A. , & Villarreal Saucedo, R. V. (2017b). New and interesting Laboulbeniales from Panama and neighboring areas. Nova Hedwigia, 105, 267–299. 10.1127/nova_hedwigia/2017/0410

[ece34359-bib-0052] Hall, T. A. (1999). BioEdit: A user‐friendly biological sequence alignment editor and analysis program for Windows 95/98/NT. Nucleic Acids Symposium Series, 41, 95–98.

[ece34359-bib-0053] Handley, C. O. Jr. (1966). Checklist of the mammals of Panama In Wenzel R. L., & Tipton V. J. (Eds.), Ectoparasites of Panama (pp. 753–795). Chicago: Field Museum of Natural History.

[ece34359-bib-0054] Handley, C. O. Jr. (1981). Key to the bats of the lowlands of Panama. Washington DC: National Museum of Natural History.

[ece34359-bib-0055] Hosokawa, T. , Nikoh, N. , Koga, R. , Satô, M. , Tanahashi, M. , Meng, X. Y. , & Fukatsu, T. (2012). Reductive genome evolution, host‐symbiont co‐speciation and uterine transmission of endosymbiotic bacteria in bat flies. The ISME Journal, 6, 577–587. 10.1038/ismej.2011.125 21938025 PMC3280136

[ece34359-bib-0056] Index Fungorum . (2018). Search Index Fungorum. Available at http://www.indexfungorum.org/names/Names.asp (accessed 15 May 2018).

[ece34359-bib-0059] Jung, S. , Kim, H. , Yamada, K. , & Lee, S. (2010). Molecular phylogeny and evolutionary habitat transition of the flower bugs (Heteroptera: Anthocoridae). Molecular Phylogenetics and Evolution, 57, 1173–1183. 10.1016/j.ympev.2010.09.013 20888925

[ece34359-bib-0062] Kolenati, F. A. (1857). Epizoa der Nycteribien. Wiener Entomologische Monatsschrift, 1, 66–69.

[ece34359-bib-0063] Kumar, S. , Stecher, G. , & Tamura, K. (2016). MEGA7: Molecular evolutionary genetics analysis version 7.0 for bigger datasets. Molecular Biology and Evolution, 33, 1870–1874. 10.1093/molbev/msw054 27004904 PMC8210823

[ece34359-bib-0066] Kunz, T. H. , & Lumsdem, L. F. (2003). Ecology of cavity and foliage roosting bats In Kunz T. H., & Fenton M. B. (Eds.), Bat ecology (pp. 3–90). Chicago: The University of Chicago Press.

[ece34359-bib-0069] Lourenço, E. C. , Almeida, J. C. , & Famadas, K. M. (2016). Richness of ectoparasitic flies (Diptera: Streblidae) of bats (Chiroptera)—a systematic review and meta‐analysis of studies in Brazil. Parasitology Research, 115, 4379–4388. 10.1007/s00436-016-5223-y 27503189

[ece34359-bib-0072] Marshall, A. G. (1981). The ecology of ectoparasitic insects. New York: Academic Press.

[ece34359-bib-0073] Merola, A. (1952). Interessante ritrovamento di labulbeniologia cavernicola: *Arthrorhynchus acrandros* n. sp. (con considerazioni sul gen. *Arthrorhynchus*). Bollettino della Società dei naturalisti in Napoli, 60, 1–30.

[ece34359-bib-0075] Miadlikowska, J. , & Lutzoni, F. (2000). Phylogenetic revision of the genus *Peltigera* (lichen‐forming Ascomycota) based on morphological, chemical, and large subunit nuclear ribosomal DNA data. International Journal of Plant Sciences, 161, 925–958. 10.1086/317568

[ece34359-bib-0077] Miller, M. A. , Pfeiffer, W. , & Schwartz, T. (2010). Creating the CIPRES Science Gateway for inference of large phylogenetic trees In Proceedings of the Gateway Computing Environments Workshop (GCE) 14 Nov. 2010, pp. 1–8. New Orleans, Louisiana.

[ece34359-bib-0078] Miller, J. , & Tschapka, M. (2001). The bat flies of La Selva (Diptera: Nycteribiidae, Streblidae). Available at http://www.biologie.uni-ulm.de/bio3/Batfly/index.html (accessed 04 November 2017).

[ece34359-bib-0079] Moras, L. M. , Gregorin, R. , Sattler, T. , & Tavares, V. C. (2018). Uncovering the diversity of dog‐faced bats of the genus *Cynomops* (Chiroptera: Molossidae), with the redescription of *C. milleri* and the description of two new species. Mammalian Biology, 89, 37–51. 10.1016/j.mambio.2017.12.005

[ece34359-bib-0080] Morse, S. F. , Bush, S. E. , Patterson, B. D. , Dick, C. W. , Gruwell, M. E. , & Dittmar, K. (2013). Evolution, multiple acquisition, and localization of endosymbionts in bat flies (Diptera: Hippoboscoidea: Streblidae and Nycteribiidae). Applied and Environmental Microbiology, 79, 2952–2961. 10.1128/AEM.03814-12 23435889 PMC3623134

[ece34359-bib-0081] Morse, S. F. , Dick, C. W. , Patterson, B. D. , & Dittmar, K. (2012). Some like it hot–Evolution and ecology of novel endosymbionts in bat flies of cave‐roosting bats (Hippoboscoidea, Nycterophiliinae). Applied and Environmental Microbiology, 78, 8639–8649. 10.1128/AEM.02455-12 23042170 PMC3502899

[ece34359-bib-0083] Olival, K. J. , Dick, C. W. , Simmons, N. B. , Morales, J. C. , Melnick, D. J. , Dittmar, K. , … DeSalle, R. (2013). Lack of population genetic structure and host specificity in the bat fly, *Cyclopodia horsfieldi*, across species of Pteropus bats in Southeast Asia. Parasites & Vectors, 6, 231 10.1186/1756-3305-6-231 23924629 PMC3750525

[ece34359-bib-0084] Overal, W. L. (1980). Host‐relations of the batfly *Megistopoda aranea* (Diptera: Streblidae) in Panama. University of Kansas Science Bulletin, 52, 1–20.

[ece34359-bib-0085] Palmeirim, J. M. , & Etherdige, K. (1985). The influence of man‐made trails on foraging by tropical frugivorous bats. Biotropica, 17, 82–83. 10.2307/2388385

[ece34359-bib-0086] Paradis, E. , Claude, J. , & Strimmer, K. (2004). APE: Analyses of phylogenetics and evolution in R language. Bioinformatics, 20, 289–290. 10.1093/bioinformatics/btg412 14734327

[ece34359-bib-0087] Parratt, S. R. , Barrès, B. , Penczykowski, R. M. , & Laine, A. L. (2017). Local adaptation at higher trophic levels: Contrasting hyperparasite–pathogen infection dynamics in the field and laboratory. Molecular Ecology, 26, 1964–1979. 10.1111/mec.13928 27859910 PMC5412677

[ece34359-bib-0088] Parratt, S. R. , & Laine, A. L. (2016). The role of hyperparasitism in microbial pathogen ecology and evolution. The ISME Journal, 10, 1815–1822. 10.1038/ismej.2015.247 26784356 PMC5029149

[ece34359-bib-0089] Patterson, B. D. , Dick, C. W. , & Dittmar, K. (2007). Roosting habits of bats affect their parasitism by bat flies (Diptera: Streblidae). Journal of Tropical Ecology, 23, 177–189. 10.1017/S0266467406003816

[ece34359-bib-0090] Peyritsch, J. (1871). Über einige Pilze aus der Familie der Laboulbenien. Sitzungsberichte der Kaiserlichen Akademie der Wissenschaften. Mathematisch‐naturwissenschaftliche Classe, 64, 441–458.

[ece34359-bib-0091] Peyritsch, J. (1873). Beiträge zur Kenntniss der Laboulbenien. Sitzungsberichte der Kaiserlichen Akademie der Wissenschaften. Mathematisch‐naturwissenschaftliche Classe, 68, 227–254.

[ece34359-bib-0092] Pfliegler, W. P. , Báthori, F. , Haelewaters, D. , & Tartally, A. (2016). Studies of Laboulbeniales on *Myrmica* ants (III): Myrmecophilous arthropods as alternative hosts of *Rickia wasmannii* . Parasite, 23, 50 10.1051/parasite/2016060 27849516 PMC5112767

[ece34359-bib-0093] Pilosof, S. , Dick, C. W. , Korine, C. , Patterson, B. D. , & Krasnov, B. R. (2012). Effects of anthropogenic disturbance and climate on patterns of bat fly parasitism. PLoS ONE, 7, e41487 10.1371/journal.pone.0041487 22829953 PMC3400619

[ece34359-bib-0095] R Core Team . (2013). R: A language and environment for statistical computing. Vienna, Austria: R Foundation for Statistical Computing Available at http://www.R-project.org (accessed 06 March 2018).

[ece34359-bib-0096] Ramasindrazana, B. , Goodman, S. M. , Gomard, Y. , Dick, C. W. , & Tortosa, P. (2017). Hidden diversity of Nycteribiidae (Diptera) bat flies from the Malagasy region and insights on host‐parasite interactions. Parasites & Vectors, 10, 630 10.1186/s13071-017-2582-x 29284533 PMC5747079

[ece34359-bib-0097] Rambaut, A. , Suchard, M. A. , Xie, D. , & Drummond, A. J. (2014). Tracer v1.6. Available at http://tree.bio.ed.ac.uk/software/tracer/ (accessed 02 November 2017).

[ece34359-bib-0098] Reboleira, A. S. P. S. , Fresneda, J. , & Salgado, J. M. (2017). A new species of *Speonemadus* from Portugal, with the revision of the *escalerai*‐group (Coleoptera, Leiodidae). European Journal of Taxonomy, 261, 1–23.

[ece34359-bib-0099] Rosenblum, E. B. , Sarver, B. A. J. , Brown, J. W. , Des Roches, S. , Hardwick, K. M. , Hether, T. D. , … Harmon, L. J. (2012). Goldilocks meets Santa Rosalia: An ephemeral speciation model explains patterns of diversification across time scales. Evolutionary Biology, 39, 255–261. 10.1007/s11692-012-9171-x 22707806 PMC3364415

[ece34359-bib-0100] Rossi, W. , & Kirk‐Spriggs, A. H. (2011). A new species of *Laboulbenia* (Ascomycota) parasitic on an African fly (Diptera: Curtonotidae), with a brief review of Diptera‐associated species of the genus. African Invertebrates, 52, 211–216. 10.5733/afin.052.0111

[ece34359-bib-0101] Rossi, W. , & Leonardi, M. (2013). New species of *Stigmatomyces* (Laboulbeniomycetes) from Sierra Leone. Plant Biosystems, 147, 79–83. 10.1080/11263504.2012.695297

[ece34359-bib-0102] Saitou, N. , & Nei, M. (1987). The neighbor‐joining method: A new method for reconstructing phylogenetic trees. Molecular Biology and Evolution, 4, 406–425. 3447015 10.1093/oxfordjournals.molbev.a040454

[ece34359-bib-0506] Samudio Jr, R. (2002). Patterns of diversity and ecology in Panamanian bats at two elevations. PhD dissertation, University of Florida, Gainesville.

[ece34359-bib-0103] Samudio, R. Jr. , & Pino, J. L. (2014). Historia de la Mastozoología en Panamá In Ortega J., Martínez J. L., & Tirira D. G. (Eds.), Historia de La Mastozoología En Latinoamérica, Las Guayanas Y El Caribe (pp. 329–344). Quito, Ecuador: Editorial Murciélago Blanco y Asociación Ecuatoriana de Mastozoología.

[ece34359-bib-0104] Santamaria, S. (1995). *Parvomyces*, a new genus of Laboulbeniales from Spain. Mycological Research, 99, 1071–1077. 10.1016/S0953-7562(09)80775-2 18554887

[ece34359-bib-0105] Santamaria, S. , Balazuc, J. , & Tavares, I. I. (1991). Distribution of the European Laboulbeniales (Fungi, Ascomycotina). An annotated list of species. Treballs de l'Institut Botanic de Barcelona, 14, 1–123.

[ece34359-bib-0106] Schuh, R. T. , & Slater, J. A. (1995). True bugs of the world (Hemiptera: Heteroptera): Classification and natural history. Ithaca, New York: Cornell University Press.

[ece34359-bib-0107] Schuh, R. T. , & S̆tys, P. (1991). Phylogenetic analysis of cimicomorphan family relationships (Heteroptera). Journal of the New York Entomological Society, 99, 298–350.

[ece34359-bib-0507] Shockley, F. W. , & Murray, K. L. (2006). Discovery of a parasitoid and a predator of bat flies (Diptera: Streblidae) at La Selva, Costa Rica. Biotropica, 38, 789–790. 10.1111/j.1744-7429.2006.00207.x

[ece34359-bib-0109] Simmons, N. B. (2005). Order Chiroptera In Wilson D. E., & Reeder D. A. M. (Eds.), Mammal species of the world: A taxonomic and geographic reference, 3rd ed. (pp. 312–529). Baltimore, Maryland: Johns Hopkins University Press.

[ece34359-bib-0110] Stadler, T. (2009). On incomplete sampling under birth–death models and connections to the sampling‐based coalescent. Journal of Theoretical Biology, 261, 58–66. 10.1016/j.jtbi.2009.07.018 19631666

[ece34359-bib-0111] Stamatakis, A. (2014). RAxML Version 8: A tool for phylogenetic analysis and post‐analysis of large phylogenies. Bioinformatics, 30, 1312–1313. 10.1093/bioinformatics/btu033 24451623 PMC3998144

[ece34359-bib-0112] Stamper, E. (2012). Host Specificity of Ecuadorian bat flies (Diptera: Streblidae). Honors College Capstone Experience/Thesis Projects. Paper 358. Available at http://digitalcommons.wku.edu/stu_hon_theses/358 (accessed 17 October 2017).

[ece34359-bib-0113] Swofford, D. L. (1991). PAUP: Phylogenetic Analysis Using Parsimony, Version 3.1. Computer program distributed by the Illinois Natural History Survey, Champaign, Illinois.

[ece34359-bib-0114] Szentiványi, T. , Haelewaters, D. , Pfliegler, W. P. , Clément, L. , Christe, P. , & Glaizot, O. (2018). Laboulbeniales (Fungi: Ascomycota) infection of bat flies (Diptera: Nycteribiidae) from *Miniopterus schreibersii* across Europe. Parasites & Vectors, 11, 395 10.1186/s13071-018-2921-6 29976258 PMC6034341

[ece34359-bib-0115] Tavares, I. I. (1985). Laboulbeniales (Fungi, Ascomycetes). Mycol Memoir, 9, 1–627.

[ece34359-bib-0116] Tavares, I. I. , & Balazuc, J. (1989). *Sugiyamaemyces*, a new genus of Laboulbeniales (Ascomycetes) on Clidicus (Scydmaenidae). Mycotaxon, 34, 565–576.

[ece34359-bib-0118] ter Hofstede, H. M. , Fenton, M. B. , & Whitaker, J. O. Jr. (2004). Host and host‐site specificity of bat flies (Diptera: Streblidae and Nycteribiidae) on Neotropical bats (Chiroptera). Canadian Journal of Zoology, 82, 616–626. 10.1139/z04-030

[ece34359-bib-0119] Thaxter, R. (1896). Contribution towards a monograph of the Laboulbeniaceae. Memoirs of the American Academy of Arts and Sciences, 12, 187–429.

[ece34359-bib-0120] Thaxter, R. (1901). Preliminary diagnosis of new species of Laboulbeniaceae. III. Proceedings of the American Academy of Arts and Sciences, 36, 397–414. 10.2307/20021044

[ece34359-bib-0121] Thaxter, R. (1908). Contribution toward a monograph of the Laboulbeniaceae. Part II. Memoirs of the American Academy of Arts and Sciences, 13, 217–469.

[ece34359-bib-0122] Thaxter, R. (1915). New Indo‐Malayan Laboulbeniales. Proceedings of the American Academy of Arts and Sciences, 51, 3–51. 10.2307/20025560

[ece34359-bib-0123] Thaxter, R. (1917). New Laboulbeniales, chiefly dipterophilous American species. Proceedings of the American Academy of Arts and Sciences, 52, 649–721. 10.2307/20025703

[ece34359-bib-0124] Thaxter, R. (1924). Contribution toward a monograph of the Laboulbeniaceae III. Memoirs of the American Academy of Arts and Sciences, 14, 309–426.

[ece34359-bib-0125] Thaxter, R. (1931). Contribution toward a monograph of the Laboulbeniaceae V. Memoirs of the American Academy of Arts and Sciences, 16, 1–435. 10.2307/25058136

[ece34359-bib-0127] Timm, R. M. , & LaVal, R. K. (1998). A field key to the bats of Costa Rica. Occasional Publication Series, University of Kansas Center of Latin American Studies, 22, 1–30.

[ece34359-bib-0128] Velazco, P. M. (2005). Morphological phylogeny of the bat genus *Platyrrhinus* Saussure, 1860 (Chiroptera: Phyllostomidae) with the description of four new species. Fieldiana: Zoology, 105, 1–53.

[ece34359-bib-0129] Vilgalys, R. , & Hester, M. (1990). Rapid genetic identification and mapping of enzymatically amplified ribosomal DNA from several *Cryptococcus* species. Journal of Bacteriology, 172, 4238–4246. 10.1128/jb.172.8.4238-4246.1990 2376561 PMC213247

[ece34359-bib-0131] Walker, M. J. , Dorrestein, A. , Camacho, J. J. , Meckler, L. A. , Silas, K. A. , Hiller, T. , & Haelewaters, D. (2018). A tripartite survey of hyperparasitic fungi associated with ectoparasitic flies on bats (Mammalia: Chiroptera) in a neotropical cloud forest in Panama. Parasite, 25, 19 10.1051/parasite/2018017 29633707 PMC5892177

[ece34359-bib-0132] Weir, A. (2008). The genus *Prolixandromyces* (Laboulbeniales) in the old world. Aliso, 26, 29–35. 10.5642/aliso

[ece34359-bib-0133] Weir, A. , & Blackwell, M. (2001a). Molecular data support the Laboulbeniales as a separate class of Ascomycota, Laboulbeniomycetes. Mycological Research, 105, 1182–1190. 10.1016/S0953-7562(08)61989-9

[ece34359-bib-0134] Weir, A. , & Blackwell, M. (2001b). Extraction and PCR amplification of DNA from minute ectoparasitic fungi. Mycologia, 93, 802–806. 10.2307/3761835

[ece34359-bib-0135] Weir, A. , & Hammond, P. M. (1997). Laboulbeniales on beetles: Host utilization patterns and species richness of the parasites. Biodiversity and Conservation, 6, 701–719. 10.1023/A:1018318320019

[ece34359-bib-0136] Wenzel, R. L. (1976). The streblid batflies of Venezuela (Diptera: Streblidae). Brigham Young University Science Bulletin, 20, 1–177.

[ece34359-bib-0137] Wenzel, R. L. , & Tipton, V. J. (1966). Ectoparasites of Panama. Chicago: Field Museum of Natural History.

[ece34359-bib-0138] Wenzel, R. L. , Tipton, V. J. , & Kiewlicz, A. (1966). The streblid batflies of Panama (Diptera Calypterae: Streblidae) In Wenzel R. L., & Tipton V. J. (Eds.), Ectoparasites of Panama (pp. 405–676). Chicago: Field Museum of Natural History.

[ece34359-bib-0139] West‐Eberhard, M. J. (1989). Phenotypic plasticity and the origins of diversity. Annual Review of Ecology, Evolution, and Systematics, 20, 249–278. 10.1146/annurev.es.20.110189.001341

[ece34359-bib-0140] Whisler, H. C. (1968). Experimental studies with a new species of *Stigmatomyces* (Laboulbeniales). Mycologia, 60, 65–75. 10.2307/3757314 5644960

[ece34359-bib-0141] Wilkinson, D. A. , Duron, O. , Cordonin, C. , Gomard, Y. , Ramasindrazana, B. , Mavingui, P. , … Tortosa, P. (2016). The bacteriome of bat flies (Nycteribiidae) from the Malagasy region: A community shaped by host ecology, bacterial transmission mode, and host‐vector specificity. Applied and Environmental Microbiology, 82, 1778–1788. 10.1128/AEM.03505-15 26746715 PMC4784053

[ece34359-bib-0142] Windsor, D. A. (1990). Heavenly hosts. Nature, 348, 104 10.1038/348104c0 2234071

[ece34359-bib-0143] Windsor, D. A. (1995). Equal rights for parasites. Conservation Biology, 9, 1–2. 10.1046/j.1523-1739.1995.09010001.x 9058952

[ece34359-bib-0144] Wrzosek, M. (2000). Taksonomia i filogeneza Mucorales (Zygomycetes) w świetle analiz morfometrycznych oraz wybranych markerów molekularnych. [Taxonomy and phylogeny of Mucorales (Zygomycetes) in the light of morphometrical and selected molecular markers analyses]. PhD dissertation, University of Warsaw.

[ece34359-bib-0145] Yule, G. U. (1925). A mathematical theory of evolution based on the conclusions of Dr. J. C. Willis. Philosophical Transactions of the Royal Society B, 213, 21–87. 10.1098/rstb.1925.0002

